# Safety, Reactogenicity, Immunogenicity, and Dose Selection of 10-Valent Extraintestinal Pathogenic *Escherichia coli* Bioconjugate Vaccine (VAC52416) in Adults Aged 60–85 Years in a Randomized, Multicenter, Interventional, First-in-Human, Phase 1/2a Study

**DOI:** 10.1093/ofid/ofad417

**Published:** 2023-08-11

**Authors:** Carlos A Fierro, Michal Sarnecki, Joachim Doua, Bart Spiessens, Oscar Go, Todd A Davies, Germie van den Dobbelsteen, Jan Poolman, Darren Abbanat, Wouter Haazen

**Affiliations:** Johnson County Clin-Trials, Lenexa, Kansas, USA; Infectious Diseases and Vaccines, Janssen Research and Development, Janssen Vaccines, Bern, Switzerland; Infectious Diseases and Vaccines, Janssen Research and Development, Janssen Pharmaceutica, Beerse, Belgium; Infectious Diseases and Vaccines, Janssen Research and Development, Janssen Pharmaceutica, Beerse, Belgium; Janssen Research and Development, Raritan, New Jersey, USA; Janssen Research and Development, Raritan, New Jersey, USA; Bacterial Vaccines Discovery and Early Development, Janssen Vaccines and Prevention B.V., Leiden, The Netherlands; Bacterial Vaccines Discovery and Early Development, Janssen Vaccines and Prevention B.V., Leiden, The Netherlands; Janssen Research and Development, Raritan, New Jersey, USA; Infectious Diseases and Vaccines, Janssen Research and Development, Janssen Pharmaceutica, Beerse, Belgium

**Keywords:** *E coli*, bacteremia, extraintestinal pathogenic *E coli*, invasive *E coli* disease, vaccine

## Abstract

**Background:**

ExPEC10V is a bioconjugate vaccine containing O-antigen polysaccharides of 10 extraintestinal pathogenic *Escherichia coli* (ExPEC) serotypes. This phase 1/2a study (NCT03819049) assessed the safety, reactogenicity, and immunogenicity of ExPEC10V (VAC52416) to prevent invasive *E coli* disease in elderly adults.

**Methods:**

The observer-blind, active-controlled design included a 28-day screening, vaccination, 181-day follow-up, and 1-year follow-up. Participants (60–85 years of age) were randomized to ExPEC10V low dose (antigen dose range, 4–8 µg), ExPEC10V medium dose (4–16 µg), or ExPEC10V high dose (8–16 µg); 4-valent ExPEC vaccine (ExPEC4V); or 13-valent pneumococcal conjugate vaccine (PCV13). The incidence of adverse events (AEs; solicited, day 15; unsolicited, day 30; serious AEs, day 181) and immunogenicity (electrochemiluminescent-based assay [ECL] and multiplex opsonophagocytic assay [MOPA]) were assessed. Optimal ExPEC10V dose was determined from safety data through day 30 and an immunogenicity dose selection algorithm based on day 15 ECL and MOPA results.

**Results:**

A total of 416 participants were included (median age, 64.0 years; 54.8% female). The incidences of solicited local and systemic AEs were, respectively, 44.2% and 39.4% for low-dose, 52.9% and 46.1% for medium-dose, 57.7% and 45.2% for high-dose ExPEC10V, and 74.1% and 48.1% for PCV13. Five serious AEs, not vaccine related, were reported. The ECL revealed a robust antibody response to ExPEC10V through year 1. Opsonophagocytic killing activity was detected against all but serotype O8; this lack of response against serotype O8 was linked to low assay sensitivity. Based on the totality of data, high-dose ExPEC10V was considered optimal.

**Conclusions:**

ExPEC10V was well tolerated and immunogenic in elderly adults against all but serotype O8.

## Teaser

A 10-valent extraintestinal pathogenic *Escherichia coli* (ExPEC) vaccine was well tolerated and demonstrated a robust functional antibody response against all but 1 serotype. The high dose was selected as optimal, and a reformulated 9-valent ExPEC vaccine is in development as a potential prophylactic vaccine against invasive ExPEC disease.

Extraintestinal pathogenic *Escherichia coli* (ExPEC) is the most common gram-negative pathogen in humans [1–3]. ExPEC is a leading cause of infections of the bloodstream or other normally sterile body sites, including intra-abdominal infections [1, 3, 4]. Invasive *E coli* disease, also known as invasive ExPEC disease (IED), can be defined as a bacterial infection with acute systemic consequences, based on clinical criteria and microbiological confirmation by the isolation and identification of *E coli* from a normally sterile body site (including blood), or from urine in patients with urosepsis and no other identifiable source of infection [5, 6]. IED is a rising cause of bacteremia, sepsis, septic shock, and death [1, 7–15].

*Escherichia coli* is responsible for the majority of community-acquired, hospital-acquired, and healthcare-associated clinically significant bloodstream infections [16]. Incidence of bacteremic IED increases with age and is most common in persons ≥60 years [10, 16–19]. Increasing antimicrobial resistance of ExPEC strains is contributing to a rising incidence and economic burden of IED [20, 21]. *Escherichia coli* has been identified as a leading antibiotic-resistant pathogen implicated in global deaths attributable to and associated with antibiotic resistance [21, 22]. The estimated case fatality rate for all IED is between 12.4% and 18.4% [13, 14]. As such, there is an urgent need for a prophylactic vaccine to prevent IED.

VAC52416 (ExPEC10V) capitalizes upon the glycoconjugate technology that was successfully used to develop vaccines against pneumococcal and meningococcal bacteremia and meningitis [23, 24] and has shown promise for the development of a licensed *Shigella* vaccine [25]. ExPEC10V is a 10-valent vaccine targeting the surface lipopolysaccharide O-antigens of ExPEC. The vaccine is comprised of 10 O-serotypes (O1A, O2, O4, O6A, O8, O15, O16, O18A, O25B, and O75) separately bioconjugated to a detoxified form of the carrier protein exotoxin A derived from *Pseudomonas aeruginosa* (EPA). The selected serotypes were found to be among the most prevalent ExPEC O-serotypes causing bloodstream infections in elderly adults across multiple geographic regions and were associated with approximately 67.5% of all ExPEC bacteremia cases analyzed with some regional variation [26]. The low, medium, and high doses of ExPEC10V were selected for evaluation based on the immunogenicity and safety results of a previous ExPEC4V (4-valent) phase 2 study, where 4:4:4:8 and 8:8:8:16 µg polysaccharide doses (serotypes O1A, O2, O6A, and O25B, respectively) produced serotype-specific antibody responses through 1 year [27]. ExPEC4V was well tolerated and immunogenic across several studies [23, 27, 28].

The objective of this phase 1/2a study (NCT03819049) was to assess the safety, reactogenicity, and immunogenicity of 3 doses of ExPEC10V as a potential vaccine candidate to prevent IED. The study included 2 cohorts. In addition to the safety evaluation, cohort 1 was used to select the optimal dose of ExPEC10V for further characterization in cohort 2. Here, we describe results from cohort 1 through year 1 of follow-up.

## METHODS

### Study Design

This was a randomized, multicenter, interventional, phase 1/2a study (NCT03819049). Cohort 1 was conducted across 6 sites in the United States and aimed to assess safety, reactogenicity, and immunogenicity of ExPEC10V and to select the optimal dose. Cohort 2 was conducted across 27 sites in the United States and Europe and aimed to characterize safety and immunogenicity of the optimal dose of ExPEC10V (selected from the analysis of cohort 1) in participants with a history of urinary tract infection (UTI; within the past 5 years). The study was initiated on 13 June 2019. The cutoff date for this analysis was 27 October 2021.

### Patient Consent Statement

This study was reviewed and approved by the institutional review board and/or independent ethics committee of each study site before the start of the study. All procedures were conducted in accordance with the ethical principles of the Declaration of Helsinki and are consistent with Good Clinical Practice. All participants provided written informed consent.

### Study Participants and Procedures

Healthy adults aged ≥60 and ≤85 years were enrolled in cohort 1 (for the full inclusion and exclusion criteria, see Supplementary Table 1). Females were required to be postmenopausal or not intending to conceive. All participants were required to have a body mass index (BMI) >18.5 and <40 kg/m^2^. Participants with an acute illness or history of an underlying clinically significant medical condition for which participation was considered not in the best interest of the participant were excluded based on the investigators’ judgment. Participants were also excluded from cohort 1 if they had previously received the 13-valent pneumococcal conjugate vaccine (PCV13) or other pneumococcal conjugate vaccines (PCVs) or were planned to receive any PCV before the final analysis. Participants either received the polyvalent pneumococcal polysaccharide vaccine (PPSV23) ≥1 year before screening or did not receive PPSV23 before the final analysis. Concomitant therapies were permitted and recorded.

An observer-blind, active-controlled design was used. There was a maximum 28-day screening period and an observer-blind 181-day follow-up period with vaccination on day 1. Participants were randomly assigned to receive a single dose of 1 of 5 vaccinations: a low dose of ExPEC10V, a medium dose of ExPEC10V, or a high dose of ExPEC10V; a single dose of ExPEC4V; or a single dose of PCV13 (Table 1). Low-dose ExPEC10V contained 4 µg of each of ExPEC O*-*serotypes O1A, O2, O4, O6A, O8, O15, O16, O18A, O75, and 8 µg of O25B. Medium-dose ExPEC10V contained 4 µg of O2, O4, O8, O15, O16, O18A, O75; 8 µg of O1A and O6A; and 16 µg of O25B. High-dose ExPEC10V contained 8 µg of O1A, O2, O4, O6A, O8, O15, O16, O18A, O75, and 16 µg of O25B (Table 1). ExPEC4V and PCV13 served as active controls. Participants who received ExPEC10V at the dose considered optimal and those who received PCV13 progressed to an open-label, long-term follow-up from day 182 through 5 years postvaccination.

**Table 1.** Extraintestinal Pathogenic *Escherichia coli* Study Vaccination

Study Vaccination Group	O1A	O2	O4	O6A	O8	O15	O16	O18A	O25B	O75	EPA	PS (Total)
Low-dose ExPEC10V	4	4	4	4	4	4	4	4	8	4	159	44
Medium-dose ExPEC10V	8	4	4	8	4	4	4	4	16	4	217	60
High-dose ExPEC10V	8	8	8	8	8	8	8	8	16	8	319	88
ExPEC4V	4	4	…	4	…	…	…	…	8	…	72	20

Data are presented as micrograms (μg). ExPEC4V consisted of the O-antigen PSs of the ExPEC serotypes O1A, O2, O6A, and O25B separately bioconjugated to the EPA carrier protein. ExPEC10V consisted of the O-antigen PSs of the ExPEC serotypes O1A, O2, O4, O6A, O8, O15, O16, O18A, O25B, and O75 separately bioconjugated to the EPA carrier protein. EPA (μg) was calculated using a ratio of 0.276 for PS/EPA. However, the final EPA dose was confirmed at the release.

Abbreviations: EPA, genetically detoxified form of exotoxin A derived from *Pseudomonas aeruginosa*; ExPEC4V, 4-valent extraintestinal pathogenic *Escherichia coli* vaccine; ExPEC10V, 10-valent extraintestinal pathogenic *Escherichia coli* vaccine; PS, polysaccharide.

Participants in cohort 1 were randomized in 2 phases. In phase 1, 84 participants were enrolled in a 6-step, staggered, stepwise dose-escalating procedure, dependent on safety evaluations conducted by an internal data review committee (Supplementary Table 2). Based on acceptable safety and reactogenicity, the remaining 320 participants were randomized to receive 1 of the 3 doses of ExPEC10V, ExPEC4V, or PCV13 in a 2:2:2:1:1 ratio. All participants were centrally assigned to randomized study vaccination using an interactive web response system. Participants, clinical staff, investigators, and sponsor personnel were blinded to vaccination group allocation, except for the designated pharmacist or staff member with primary responsibility for vaccine preparation and administration. All participants randomized to any of the 3 doses of ExPEC10V or ExPEC4V were offered PCV after the final analysis database lock (day 181), in accordance with Advisory Committee for Immunization Practices recommendations.

A study evaluating cohort 2 used a double-blind, placebo-controlled design and will be reported separately.

### Study Drug

All vaccinations were administered as a single 0.5-mL intramuscular injection to the deltoid muscle (Table 1). ExPEC10V is an *E coli* bioconjugate vaccine in phosphate-buffered solution containing O-antigen polysaccharides of ExPEC serotypes O1A, O2, O4, O6A, O8, O15, O16, O18A, O25B, and O75. ExPEC4V is an *E coli* bioconjugate vaccine in saline-buffered solution containing O-antigen polysaccharides of ExPEC serotypes O1A, O2, O6A, and O25B [23, 27, 28]. PCV13 is a sterile suspension of saccharides of the capsular antigens of *Streptococcus pneumoniae* serotypes 1, 3, 4, 5, 6A, 6B, 7F, 9V, 14, 18C, 19A, 19F, and 23F, individually linked to nontoxic diphtheria CRM197 protein [29].

### Assessments

#### Safety and Reactogenicity

Primary endpoints were solicited local and systemic adverse events (AEs) collected until day 15 (14 days postvaccination), unsolicited AEs collected until day 30 (29 days postvaccination), and serious AEs (SAEs) collected until day 181 (180 days postvaccination). Solicited local AEs were considered related to study vaccination by definition. Late-onset AEs were defined as AEs with time to first onset after day 5 postvaccination. SAEs related to the study vaccine/procedures collected from day 182 until the end of the study were assessed as a secondary endpoint. After day 181, only medication taken for related SAEs was reported. Physical examinations, vital sign measurements, and clinical laboratory tests were conducted. Any clinically meaningful changes were recorded as AEs. Solicited AEs occurring after day 15 were recorded as unsolicited AEs.

#### Immunogenicity

Vaccine- and serotype-specific total antibody titers, as measured via electrochemiluminescent-based immunoassay (ECL) and multiplex opsonophagocytic assay (MOPA), on day 15 were assessed as primary endpoints. Assessments on day 30, day 181, and year 1 and the correlation between the ECL and MOPA were secondary endpoints. Venous blood samples were collected prevaccination (day 1/baseline), on days 15, 30, 181, and year 1 postvaccination.

A multiplex ECL was used to determine the levels of antibodies against *E coli* vaccine serotypes and the carrier protein EPA. A microcolony platform–based MOPA, based on the assay previously used for pneumococcus [30], was used as a functional assay to evaluate the ability of antibodies to mediate opsonophagocytic killing of *E coli* vaccine serotypes (see Supplementary Methods). Clinical serum samples collected on days 1 and 15 were analyzed with a qualified MOPA and those from days 30, 181, and year 1 were analyzed with a validated MOPA; these data cannot be directly compared. The optimal dose of ExPEC10V was determined based on the safety data through day 30 and was guided by an immunogenicity dose selection algorithm based on day 15 ECL and MOPA results (see next section).

#### Statistical Analysis and Immunogenicity Dose Algorithm

A sample size of 100 participants per ExPEC10V dose group was estimated to provide 95% confidence that if no AE or SAE is observed in a group, the true incidence is ≤2.95%. The coprimary objective of the study was to evaluate the dose-dependent immunogenicity of ExPEC10V. A sample size of 94 participants per ExPEC10V dose group was estimated to provide 90% power to detect differences (1-sided α = 5%) if 2 groups differ 2-fold (0.301 on log_10_-scale) for a certain O-serotype, assuming a standard deviation (SD) of 0.7. The full analysis set (FAS) included all randomized participants with a vaccine administration documented and was considered the primary safety population. The per-protocol immunogenicity (PPI) analysis set included all randomized and vaccinated participants for whom immunogenicity data were available, excluding samples from participants with major protocol deviations expected to impact the immunogenicity outcomes. The PPI population was the primary immunogenicity population.

Descriptive statistics were used to assess safety and immunogenicity data. For immunogenicity endpoints, geometric mean titer, geometric mean ratio (ie, geometric mean [GM] fold increase from baseline), and percentage of participants with at least a 2-fold increase from baseline were used to evaluate differences between groups. The Pearson correlation coefficient between ECL and MOPA measurements was calculated.

The immunogenicity dose selection algorithm was based on an analysis of covariance model; it included the log_10_ transformation of the fold increase from baseline to day 15 as the response variable, and the dose groups and the log_10_ transformations of the baseline titer as independent variables. The immunogenicity dose selection algorithm is described in the Supplementary Materials.

## RESULTS

### Participants

**Figure 1.**
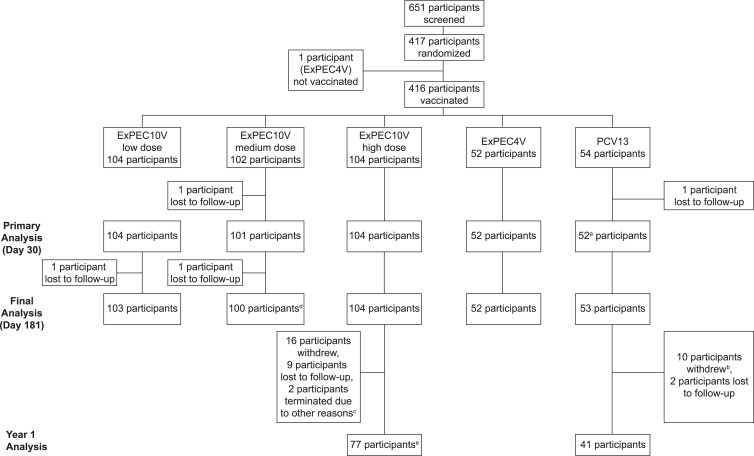
Study disposition. ^a^One participant of the 13-valent pneumococcal conjugate vaccine group did not attend the day 30 visit but did complete the day 181 visit. ^b^Among the 10 participants, 8 withdrew because they did not provide consent to participate in long-term follow-up. ^c^The 2 participants who were reported to have discontinued due to other reasons did not consent to participate in long-term follow-up. ^d^One participant did not attend the day 181 visit, but the participant replied to a certified mail on day 218. ^e^Two participants of the high-dose ExPEC10V (10-valent extraintestinal pathogenic *Escherichia coli* vaccine) group withdrew after completing the year 1 visit; 75 participants in the high-dose ExPEC10V group continued after year 1. Abbreviations: ExPEC4V, 4-valent extraintestinal pathogenic *Escherichia coli* vaccine; ExPEC10V, 10-valent extraintestinal pathogenic *Escherichia coli* vaccine; PCV13, 13-valent pneumococcal conjugate vaccine.

A total of 417 of 651 screened participants were randomized (Figure 1). One participant was inadvertently randomized to ExPEC4V after a screen failure and was not vaccinated or included in the FAS. The FAS was comprised of 416 participants who received a low (n = 104), medium (n = 102), or high (n = 104) dose of ExPEC10V, ExPEC4V (n = 52), or PCV13 (n = 54). Demographic characteristics were balanced across vaccination groups. Mean age was 65.8 (SD, 5.3) years; 54.8% of participants were female (Table 2). Most participants (89.4%) were White and of non-Hispanic ethnicity (83.9%); 9.6% were Black/African American. Mean BMI was 28.0 (SD, 3.8) kg/m^2^. Concomitant therapies were reported in 359 (86.3%) participants through day 181. The most frequently used concomitant therapies were atorvastatin (14.2%), acetylsalicylic acid (14.2%), lisinopril (12.5%), and ibuprofen (11.3%).

**Table 2.** Summary of Demographic and Baseline Characteristics

Characteristic	ExPEC10V, Low	ExPEC10V, Medium	ExPEC10V, High	ExPEC10V, Pooled	ExPEC4V	PCV13	Total
No.	104	102	104	310	52	54	416
Age, y							
Mean (SD)	66.2 (5.8)	66.4 (5.2)	65.1 (5.2)	65.9 (5.4)	64.8 (4.6)	65.9 (5.4)	65.8 (5.3)
Range	60–85	60–82	60–83	60–85	60–80	60–83	60–85
60–64 y	57 (54.8)	48 (47.1)	63 (60.6)	168 (54.2)	32 (61.5)	29 (53.7)	229 (55.0)
65–69 y	26 (25.0)	27 (26.5)	24 (23.1)	77 (24.8)	12 (23.1)	12 (22.2)	101 (24.3)
70–74 y	9 (8.7)	20 (19.6)	8 (7.7)	37 (11.9)	6 (11.5)	9 (16.7)	52 (12.5)
≥75 y	12 (11.5)	7 (6.9)	9 (8.7)	28 (9.0)	2 (3.8)	4 (7.4)	34 (8.2)
Sex							
Female	59 (56.7)	45 (44.1)	65 (62.5)	169 (54.5)	31 (59.6)	28 (51.9)	228 (54.8)
Male	45 (43.3)	57 (55.9)	39 (37.5)	141 (45.5)	21 (40.4)	26 (48.1)	188 (45.2)
Race							
White	93 (89.4)	91 (89.2)	90 (86.5)	274 (88.4)	48 (92.3)	50 (92.6)	372 (89.4)
Black/African American	10 (9.6)	10 (9.8)	13 (12.5)	33 (10.6)	3 (5.8)	4 (7.4)	40 (9.6)
Asian	0 (0.0)	0 (0.0)	1 (1.0)	1 (0.3)	0 (0.0)	0 (0.0)	1 (0.2)
Native Hawaiian/Pacific Islander	1 (1.0)	0 (0.0)	0 (0.0)	1 (0.3)	0 (0.0)	0 (0.0)	1 (0.2)
American Indian/Alaska Native	0 (0.0)	1 (1.0)	0 (0.0)	1 (0.3)	1 (1.9)	0 (0.0)	2 (0.5)
Ethnicity							
Not Hispanic or Latino	85 (81.7)	85 (83.3)	86 (82.7)	256 (82.6)	46 (88.5)	47 (87.0)	349 (83.9)
Hispanic or Latino	17 (16.3)	15 (14.7)	15 (14.4)	47 (15.2)	4 (7.7)	7 (13.0)	58 (13.9)
Not reported	2 (1.9)	2 (2.0)	3 (2.9)	7 (2.3)	2 (3.8)	0 (0.0)	9 (2.2)
BMI, kg/m^2^							
Mean (SD)	27.8 (3.9)	28.2 (3.6)	27.8 (3.9)	27.9 (3.8)	28.0 (3.9)	28.1 (3.9)	28.0 (3.8)
Range	19–35	19–35	19–35	19–35	21–35	19–35	19–35

Data are presented as No. (%) unless otherwise specified. Percentages are calculated using the total number of participants with nonmissing data as denominator.

Abbreviations: BMI, body mass index; ExPEC4V, 4-valent extraintestinal pathogenic *Escherichia coli* vaccine; ExPEC10V, 10-valent extraintestinal pathogenic *Escherichia coli* vaccine; PCV13, 13-valent pneumococcal conjugate vaccine; SD, standard deviation.

### Safety and Reactogenicity

**Figure 2.**
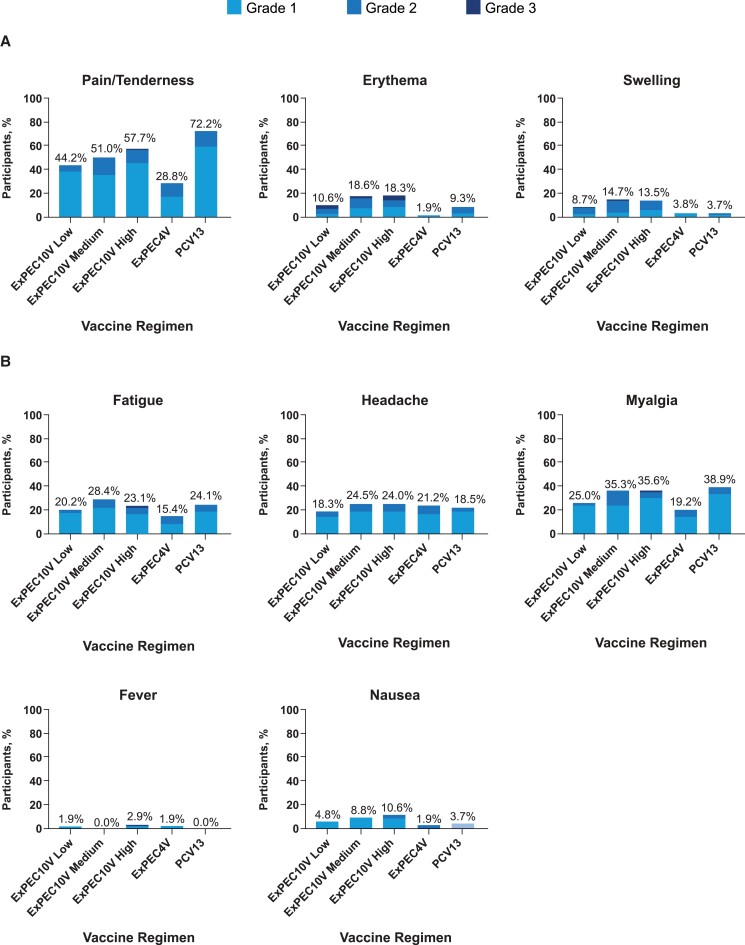
Solicited local (*A*) and systemic (*B*) adverse events (AEs) by worst severity grade. Data presented are percentage of participants with local and systemic solicited AEs from day 1 to day 15 in the full analysis set. Abbreviations: ExPEC4V, 4-valent extraintestinal pathogenic *Escherichia coli* vaccine; ExPEC10V, 10-valent extraintestinal pathogenic *Escherichia coli* vaccine; PCV13, 13-valent pneumococcal conjugate vaccine.

The incidence of local and systemic solicited and unsolicited AEs by grade is summarized in Table 3. The incidences of solicited local and systemic AEs in ExPEC10V groups were 44.2% and 39.4% for low dose, 52.9% and 46.1% for medium dose, and 57.7% and 45.2% for high dose, respectively; these were lower compared to those observed in the PCV13 group (74.1% and 48.1%, respectively). The majority of solicited local and systemic AEs were grade 1 or 2 in severity (Figure 2). The most frequently observed solicited local AE was injection site pain/tenderness. Grade 3 solicited local and systemic AEs were only observed in ExPEC10V groups (Table 3). The most frequently observed solicited systemic AEs were myalgia, fatigue, and headache. The incidence of solicited systemic AEs considered to be related to study vaccination was 37.5% for low-dose, 43.1% for medium-dose, and 42.3% for high-dose ExPEC10V; 30.8% for ExPEC4V, and 44.4% for PCV13. No grade 4 solicited local or systemic AEs were reported.

**Table 3.** Summary of Adverse Events

Study Vaccination Group	ExPEC10V, Low	ExPEC10V, Medium	ExPEC10V, High	ExPEC10V, Pooled	ExPEC4V	PCV13
No.	104	102	104	310	52	54
Solicited AEs	59 (56.7)	61 (59.8)	69 (66.3)	189 (61.0)	23 (44.2)	40 (74.1)
Solicited AEs of grade 3	3 (2.9)	2 (2.0)	6 (5.8)	11 (3.5)	0 (0.0)	0 (0.0)
Solicited local AEs^a^	46 (44.2)	54 (52.9)	60 (57.7)	160 (51.6)	15 (28.8)	40 (74.1)
Pain/tenderness	46 (44.2)	52 (51.0)	60 (57.7)	158 (51.0)	15 (28.8)	39 (72.2)
Erythema	11 (10.6)	19 (18.6)	19 (18.3)	49 (15.8)	1 (1.9)	5 (9.3)
Swelling	9 (8.7)	15 (14.7)	14 (13.5)	38 (12.3)	2 (3.8)	2 (3.7)
Solicited local AEs of grade 3	3 (2.9)	2 (2.0)	4 (3.8)	9 (2.9)	0 (0.0)	0 (0.0)
Solicited systemic AEs	41 (39.4)	47 (46.1)	47 (45.2)	135 (43.5)	17 (32.7)	26 (48.1)
Fatigue	21 (20.2)	29 (28.4)	24 (23.1)	74 (23.9)	8 (15.4)	13 (24.1)
Headache	19 (18.3)	25 (24.5)	25 (24.0)	69 (22.3)	11 (21.2)	10 (18.5)
Myalgia	26 (25.0)	36 (35.3)	37 (35.6)	99 (31.9)	10 (19.2)	21 (38.9)
Fever	2 (1.9)	0 (0.0)	3 (2.9)	5 (1.6)	1 (1.9)	0 (0.0)
Nausea	5 (4.8)	9 (8.8)	11 (10.6)	25 (8.1)	1 (1.9)	2 (3.7)
Solicited systemic AEs of grade 3	0 (0.0)	0 (0.0)	3 (2.9)	3 (1.0)	0 (0.0)	0 (0.0)
Solicited systemic AEs that are thought to be related to study vaccine	39 (37.5)	44 (43.1)	44 (42.3)	127 (41.0)	16 (30.8)	24 (44.4)
Solicited systemic AEs of at least grade 3 thought to be related to study vaccine	0 (0.0)	0 (0.0)	3 (2.9)	3 (1.0)	0 (0.0)	0 (0.0)
Any unsolicited AEs^b^	25 (24.0)	21 (20.6)	23 (22.1)	69 (22.3)	9 (17.3)	15 (27.8)
Upper respiratory tract infection	3 (2.9)	4 (3.9)	1 (1.0)	8 (2.6)	3 (5.8)	3 (5.6)
Injection site pruritis	1 (1.0)	5 (4.9)	3 (2.9)	9 (2.9)	2 (3.8)	1 (1.9)
Vaccination site erythema	2 (1.9)	0 (0.0)	2 (1.9)	4 (1.3)	0 (0.0)	1 (1.9)
Fatigue	1 (1.0)	1 (1.0)	1 (1.0)	3 (1.0)	0 (0.0)	0 (0.0)
Myalgia	1 (1.0)	3 (2.9)	2 (1.9)	6 (1.9)	0 (0.0)	0 (0.0)
Back pain	1 (1.0)	1 (1.0)	0 (0.0)	2 (0.6)	1 (1.9)	0 (0.0)
Systolic blood pressure increase	1 (1.0)	1 (1.0)	1 (1.0)	3 (1.0)	0 (0.0)	0 (0.0)
Diarrhea	1 (1.0)	0 (0.0)	4 (3.8)	5 (1.6)	1 (1.9)	0 (0.0)
Headache	1 (1.0)	1 (1.0)	2 (1.9)	4 (1.3)	0 (0.0)	0 (0.0)
Any unsolicited AEs of grade 3	2 (1.9)	3 (2.9)	2 (1.9)	7 (2.3)	0 (0.0)	3 (5.6)
Any unsolicited AEs of grade 4	1 (1.0)	0 (0.0)	0 (0.0)	1 (0.3)	0 (0.0)	0 (0.0)
Unsolicited AEs thought to be related to the study vaccine	5 (4.8)	10 (9.8)	11 (10.6)	26 (8.4)	3 (5.8)	5 (9.3)
SAE^c^	2 (1.9)	2 (2.0)	0 (0.0)	4 (1.3)	0 (0.0)	1 (1.9)

Data are presented as No. (%) unless otherwise specified. Participants are counted only once for any given event, regardless of the number of times they actually experienced the event. There were no solicited AEs of grade 4. Solicited local and systemic AEs were collected for 14 days postvaccination (from day 1 to day 15). Unsolicited AEs were collected from the administration of the study vaccine until 29 days postvaccination (from day 1 to day 30). SAEs were collected until day 181 (180 days postvaccination). SAEs related to the study vaccine/procedures were collected from day 182 until the end of the study.

Abbreviations: AE, adverse event; ExPEC4V, 4-valent extraintestinal pathogenic *Escherichia coli* vaccine; ExPEC10V, 10-valent extraintestinal pathogenic *Escherichia coli* vaccine; PCV13, 13-valent pneumococcal conjugate vaccine; SAE, serious adverse event.

a Solicited local AEs were considered related to study vaccine by definition.

b AEs by preferred term are those occurring in at least 3 participants overall.

c One low-dose ExPEC10V participant reported osteoarthritis (grade 2) on day 128, which resolved after 6 days. One low-dose ExPEC10V participant and 1 medium-dose ExPEC10V participant reported intervertebral disc protrusion (grade 2) on days 138 and 16, respectively, which resolved after 134 and 2 days. One medium-dose ExPEC10V participant experienced grade 3 nephrolithiasis on day 163, which resolved after 1 day. One PCV13 participant experienced grade 3 osteoarthritis on day 112, which resolved after 1 day. None of the SAEs were considered related to the study vaccine.

A portion of the solicited AEs that occurred were late onset (Supplementary Figure 1). The incidence of late-onset solicited local AEs was as follows: 37.0% (17/46), low-dose ExPEC10V; 38.9% (21/54), medium-dose ExPEC10V; 45.0% (27/60), high-dose ExPEC10V; 46.7% (7/15), ExPEC4V; and 5.0% (2/40), PCV13. Of ExPEC10V participants who reported erythema and swelling, 93.9% and 94.7% experienced late-onset erythema and swelling, respectively. Among ExPEC10V, ExPEC4V, and PCV13 participants with a solicited systemic AE, 28.2%, 17.6%, and 11.5% experienced late-onset solicited systemic AEs, respectively.

Incidence of unsolicited AEs was similar across vaccination groups; the most frequently reported unsolicited AEs were upper respiratory tract infection, injection site pruritis, and myalgia (Table 3). The rates of unsolicited AEs related to study vaccination were 4.8% for low-dose, 9.8% for medium-dose, and 10.6% for high-dose ExPEC10V; 5.8% for ExPEC4V, and 9.3% for PCV13. The incidence of grade 3 unsolicited AEs was 2.3% in all ExPEC10V participants and 5.6% in PCV13 participants; none were reported in ExPEC4V participants.

Five SAEs occurred, none of which were related to the study vaccine. No deaths were reported. There were no clinically meaningful findings in the vital sign measurements, physical examination assessments, or other observations related to safety. The majority of laboratory abnormalities after vaccination were grade 1 or grade 2 and occurred in ≤6% of participants.

### Immunogenicity

**Figure 3.**
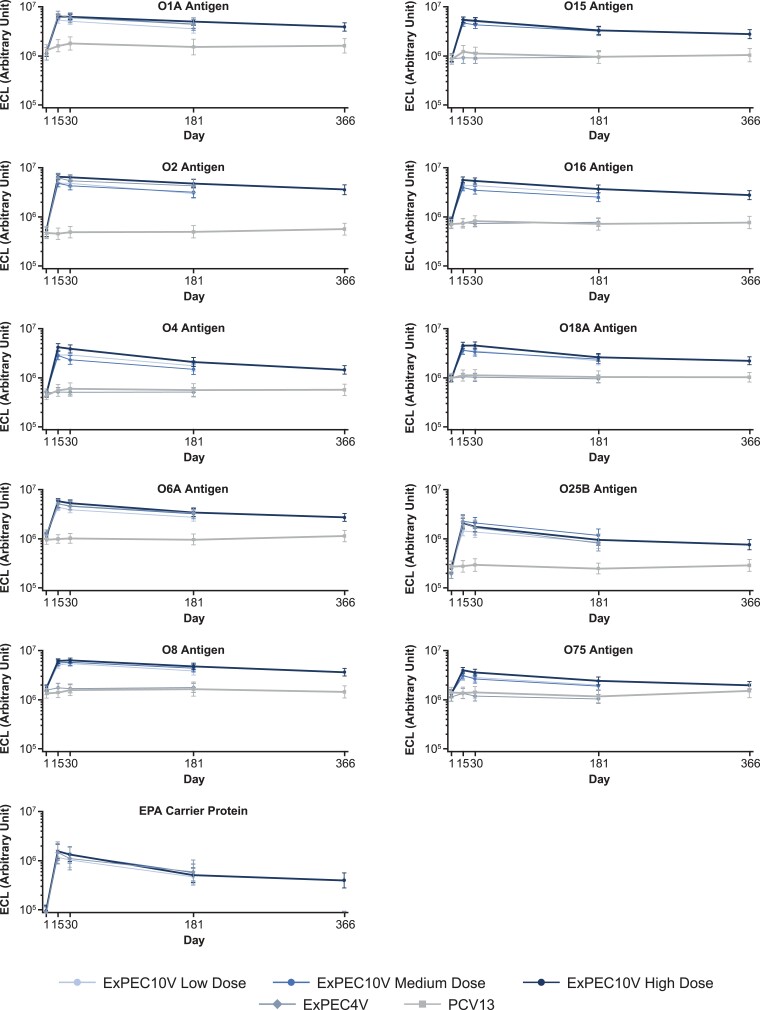
Multiplex electrochemiluminescent-based assay–determined immunoassay immunoglobulin G geometric mean titers. Data presented are from the per-protocol immunogenicity analysis set. Abbreviations: ECL, electrochemiluminescent-based assay; EPA, genetically detoxified form of exotoxin A derived from *Pseudomonas aeruginosa*; ExPEC4V, 4-valent extraintestinal pathogenic *Escherichia coli* vaccine; ExPEC10V, 10-valent extraintestinal pathogenic *Escherichia coli* vaccine; PCV13, 13-valent pneumococcal conjugate vaccine.

**Figure 4.**
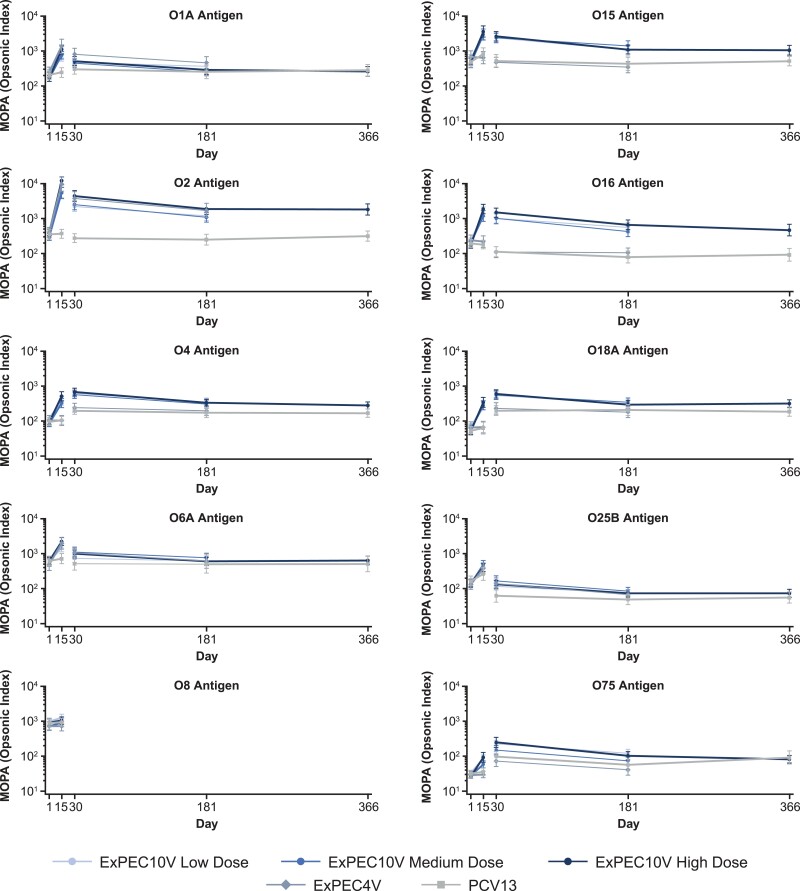
Multiplex opsonophagocytic assay (MOPA)–determined functional antibody geometric mean titers. Data presented are from the per-protocol immunogenicity analysis set. Clinical serum samples collected on days 1 and 15 were analyzed with a qualified MOPA, and those from day 30, day 181, and year 1 were analyzed with a validated assay; these data are not directly comparable. Abbreviations: ExPEC4V, 4-valent extraintestinal pathogenic *Escherichia coli* vaccine; ExPEC10V, 10-valent extraintestinal pathogenic *Escherichia coli* vaccine; MOPA, multiplex opsonophagocytic assay; PCV13, 13-valent pneumococcal conjugate vaccine.

There was a robust antibody-mediated immunogenic response to ExPEC10V against all vaccine serotypes for all 3 doses (Figures 3 and 4; Supplementary Tables 3 and 4). GM fold increases from baseline on day 15 on the ECL ranged from 2.33 to 9.54 for low-dose ExPEC10V, 2.38 to 10.05 for medium-dose ExPEC10V, and 3.06 to 12.31 for high-dose ExPEC10V participants across the 10 serotypes; 68.0%–99.0% of high-dose ExPEC10V participants exhibited at least a 2-fold increase from baseline to day 15 across serotypes.

Opsonophagocytic killing activity was demonstrated against all but serotype O8. The O8 *E coli* strain used in the assay for clinical testing was not able to discriminate a vaccine-induced immune response at baseline and day 15 (ie, the MOPA yielded similarly very high titers for both baseline and day 15 samples). Excluding O8, GM fold increase from baseline on day 15 ranged from 1.92 to 14.39 for low-dose ExPEC10V, 1.69 to 17.78 for medium-dose ExPEC10V, and 2.51 to 30.19 for high-dose ExPEC10V; 38.0%–98.0% of high-dose ExPEC10V participants exhibited at least a 2-fold increase across serotypes (excluding O8).

The highest ExPEC vaccine–mediated antibody response on day 15 was observed for the O2 serotype with both the ECL and the MOPA. Excluding serotype O8, the lowest response was observed for the O75 serotype with both the ECL and the MOPA. Minimal change in the immunogenicity response was observed between day 15 and day 30 on the ECL. The immunogenicity response decreased at day 181 and year 1 but remained above the PCV13 control group across serotypes. Coanalysis of the ECL and MOPA showed moderate (≥0.5) to strong (≥0.7) correlation between the assay responses at day 15 for the high-dose ExPEC10V group (Supplementary Figure 2). For high-dose ExPEC10V (excluding O8), Pearson correlation coefficients were 0.54–0.72, 0.45–0.71, and 0.44–0.77 on days 15, 30, and 181, respectively.

High-dose ExPEC10V exhibited the highest immunogenic responses on the ECL and MOPA and was consistently selected for further clinical development by the immunogenicity dose selection algorithm irrespective of population or assay (Supplementary Table 5).

## DISCUSSION

ExPEC10V was immunogenic and well tolerated in healthy adults 60–85 years of age. No safety signals were identified. Solicited local and systemic AEs occurred in 52% and 44% of ExPEC10V participants, respectively, with a slightly higher incidence of solicited local AEs in participants vaccinated with high-dose ExPEC10V relative to the low or medium dose. High-dose ExPEC10V participants exhibited comparable or lower rates of solicited AEs relative to the PCV13 group. The most frequent solicited local AE with ExPEC10V was injection site pain and tenderness; the most frequent solicited systemic AEs with ExPEC10V were myalgia, fatigue, and headache. Most participants who experienced erythema and swelling experienced them as late-onset AEs. However, there was no similar trend for the most common solicited systemic AEs (myalgia, fatigue, or headache). Incidence of unsolicited AEs was similar across vaccination groups, and no vaccine-related SAEs were reported.

ExPEC10V builds upon the composition of ExPEC4V, a precursor vaccine containing 4 polysaccharide O-antigens from *E coli* serotypes O1A, O2, O6A, and O25B. ExPEC4V was well tolerated and immunogenic in a range of populations, including 48 healthy Japanese participants aged ≥20 years [28], 93 healthy women with a history of recurrent UTI in Switzerland [23], and 757 healthy adult participants aged ≥18 years in the United States [27]. Robust and functional antibody responses across all 4 vaccine serotypes were observed across studies. The 10-valent formulation of ExPEC10V including additional serotypes O4, O8, O15, O16, O18, and O75 is corroborated by worldwide epidemiological data showing high prevalence of these serotypes in clinical isolates of older adults with bloodstream infections [26].

All 3 doses of ExPEC10V were immunogenic. The high dose, which was considered optimal, induced a robust increase in binding antibody titers (ECL) across all 10 serotypes, and the immunogenic response persisted above control levels through year 1. Functionality of high-dose ExPEC10V–induced serum antibodies was demonstrated through increased opsonophagocytic killing of all *E coli* vaccine serotypes except O8. The strain used in MOPA for serotype O8 was not sensitive enough to discriminate between a vaccine-induced immune response at day 15 and baseline, necessitating further assay optimization. An investigation of the lack of O8 vaccine response showed that some O8 strains lacked sensitivity to detect functional vaccine-induced antibodies while other O8 strains could detect a vaccine response. The strain used in the MOPA assay in this study was in the former category (data not shown). Different responses of strains of the same serotype in an opsonophagocytic assay have been observed for *S pneumoniae*, underscoring the importance of strain selection [31]. With removal of serotype O8 from the present vaccine composition, serotype coverage of ExPEC9V for ExPEC bacteremia across multiple geographic regions worldwide is expected to be approximately 65% (data on file). Pending further evaluations of the MOPA serotype O8 assay and the incorporation of procedural improvements, inclusion of serotype O8 may be reconsidered for future iterations of the multivalent ExPEC vaccine.

Serotype O8 was removed from the vaccine composition to expediate further clinical development. The removal of O8 allowed for an increase in the O75 polysaccharide content, which had the lowest ECL response. A reformulated 9-valent vaccine, ExPEC9V, is currently being assessed in the phase 3 trial E.mbrace (NCT04899336).

There are no established immunogenicity thresholds against which to evaluate immunogenicity results. In this study, 68.0%–99.0% and 38.0%–98.0% of participants receiving the high dose of ExPEC10V exhibited ≥2-fold increase in the serotype-specific antibody response from baseline to day 15 on ECL and MOPA, respectively. Although a ≥2 times increase in antibody titer in 80% of individuals has been proposed as a putative measure of immunogenicity for ExPEC vaccines [27], with no established immunogenicity thresholds, the present results may not be generalizable outside the United States or in participants aged <60 years or >85 years. As of the time of this report, safety data beyond 1 year postvaccination are not yet available.

In summary, data from cohort 1 through year 1 of this study provide evidence for a strong safety and immunogenicity profile of ExPEC10V and have informed the selection of the optimal dose and vaccine composition for further clinical development in cohort 2. The safety and efficacy of a reformulated 9-valent vaccine, ExPEC9V, is currently being assessed in the pivotal phase 3 trial E.mbrace (NCT04899336) as a vaccine candidate to prevent IED in adults aged 60 years and older.

## Supplementary Material

ofad417_Supplementary_Data

## Supplementary Data

Supplementary materials are available at *Open Forum Infectious Diseases* online. Consisting of data provided by the authors to benefit the reader, the posted materials are not copyedited and are the sole responsibility of the authors, so questions or comments should be addressed to the corresponding author.

## Notes

***Acknowledgments.*** Medical writing support was provided by Meghan Jones, PhD, of Eloquent Scientific Solutions, and funded by Janssen Research & Development, LLC. The authors acknowledge Kellen Fae for her contribution to this work.

***Financial support.*** This work was supported by Janssen Research & Development, LLC.
