# Supplementary material for: Safety, Reactogenicity, Immunogenicity, and Dose Selection of 10-Valent Extraintestinal Pathogenic Escherichia coli Bioconjugate Vaccine (VAC52416) in Adults Aged 60–85 Years in a Randomized, Multicenter, Interventional, First-in-Human, Phase 1/2a Study
Source: Open Forum Infect Dis. 2023 Aug 11;10(8):ofad417. doi: 10.1093/ofid/ofad417 (PMC10442062; doi:10.1093/ofid/ofad417)
Supplement: ofad417_Supplementary_Data [file ofad417_supplementary_data.zip › Fierro et al BAC1001 Revised Supplement_17JUL23_CLEAN.docx]

**SUPPLEMENTARY MATERIALS**

**METHODS**

**Immunogenicity**

A multiplex electrochemiluminescent-based assay (ECL) was used to determine the levels of immunoglobulin G (IgG) antibodies against *Escherichia coli* O1A, O2, O4, O6A, O8, O15, O16, O18A, O25B, and O75 serotypes and the carrier protein exotoxin A derived from *Pseudomonas aeruginosa* (EPA). Each assay plate contained a reference curve used to determine the relative concentration (arbitrary units/mL) of antibodies against the serotypes and EPA in the test samples. After serial dilution of the reference and clinical serum samples, the reference curve was fitted using a 4-parameter logistic regression, and the antibody-binding concentration of samples was calculated by back-fitting to the reference.

A multiplex opsonophagocytic assay (MOPA) was used as a functional assay, evaluating the ability of antibodies to mediate opsonophagocytic killing of the *E. coli* O1A, O2, O4, O6A, O8, O15, O16, O18A, O25B, and O75 serotypes. The MOPA measures the level of functional serum antibodies specific to up to 4 *E. coli* serotypes simultaneously in the same reaction multiplexing the isolates into cassettes. A microcolony platform–based MOPA similar to the methods for pneumococcus was used [1]. *E coli* strains with the serotypes that were included in the ExPEC10V vaccine were propagated based on their unique antibiotic resistance. The *E. coli* strains, organized in different cassettes, were mixed with heat-inactivated serially diluted test samples (bacterial opsonization step). Subsequently, human complement and phagocytic cells (HL-60) were added to the reaction mix (complement-mediated phagocytosis step). The reaction mix was transferred to filter plates that each contained specific antibiotics to select the resistant isolate (antibiotic selection step). The colony-forming units (CFU) were counted to determine the number of surviving bacteria. The reportable antibody titer was calculated by a linear interpolation of the CFU using points just above and just below the 50% cutoff (50% of the median of control CFU that contained bacteria, complement, and HL-60 cells but no samples [maximal bacterial growth in the assay condition]). Clinical serum samples collected on days 1 and 15 were analyzed with a qualified MOPA, and those from days 30 and 181 and year 1 were analyzed with a validated assay; these data are not directly comparable.

**Immunogenicity Dose Selection Algorithm**

The immunogenicity dose selection algorithm was based on an analysis of covariance model, which was comprised of the log_10_ transformation of the fold increase from baseline to day 15 as the response variable, and the independent variables consisting of the vaccine groups and the log_10_ transformations of the baseline titer. The algorithm implemented a stepwise procedure with the following 4 serotypes first sequentially: O25B, O6A, O2, and O1A. For each serotype, both the least squares mean (LSM) of each vaccine group included in the model and a 95% confidence interval of the difference between the LSM of a vaccine group and that vaccine group having the highest LSM were computed. The vaccine group having the highest LSM and other vaccine groups that were noninferior to this group based on the confidence limits were retained. Only these vaccine groups were included in the model as covariate in the next step. The procedure was then executed separately in a nonspecific order of serotypes for O4, O8, O15, O16, O18A, and O75. By majority rule, the vaccine group(s) that was (were) retained the most times in the 6 serotypes was the dose selected. This dose selection algorithm was performed on the full analysis set and per protocol immunogenicity analysis set and with ECL and MOPA assessments.

**Reference**

1. Nolan KM, Bonhomme ME, Schier CJ, Green T, Antonello JM, Murphy RD. Optimization and validation of a microcolony multiplexed opsonophagocytic killing assay for 15 pneumococcal serotypes. Bioanalysis **2020**; 12(14):1003-20.

**SUPPLEMENTARY TABLES**

**Supplementary Table 1. Inclusion and Exclusion Criteria**

| Inclusion Criteria |
| --- |
| Male or female, ≥60 to ≤85 years of age, inclusive, on the day of signing the informed consent form (ICF) and available for the duration of the study |
| Must have a body mass index of >18.5 to <40 kg/m^2^ |
| Must be healthy or medically stable, in the investigator’s clinical judgment, as confirmed by medical history, physical examination, vital signs, and by 12-lead electrocardiography and clinical laboratory tests, performed at the screening visit. Participant may have underlying illnesses, such as hypertension, diabetes, or ischemic heart disease, as long as their symptoms/signs are medically controlled. If the participant is on medication for a condition, the medication dose must have been stable for at least 12 weeks preceding vaccination and expected to remain stable for the duration of the study (or minor dose adaptations could be done and accepted based on physician’s judgment)  If laboratory screening tests are outside of the normal reference ranges, repeat of screening tests is permitted once (at the discretion of the investigator) during screening to assess eligibility. Enrollment of a participant with clinical laboratory values outside of the central laboratory normal range representing toxicity grade 1 or 2 is allowed if the investigator considers the values not to be clinically significant and reasonable for the population under study. This determination must be recorded in the participant’s source documents by the investigator  *Note: If laboratory screening tests are out of central laboratory normal ranges and deemed clinically significant, repeat of screening tests is permitted once, using a scheduled visit during the screening period to assess eligibility. Screening laboratory tests are to be done within 28 days of study vaccination* |
| Before randomization, a woman must be:  a. Postmenopausal (a postmenopausal state is defined as no menses for 12 months without an alternative medical cause); or  b. Not intending to conceive by any methods |
| Must sign an ICF indicating that he or she understands the purpose of, and procedures required for, the study and is willing to participate in the study |
| Willing and able to adhere to the lifestyle restrictions specified in this protocol |
| Agrees not to donate blood until 12 weeks after receiving the study vaccine |
| Must be willing to provide verifiable identification, has means to be contacted and to contact the investigator during the study |
| Exclusion Criteria |
| Must not have been previously vaccinated with PCV13 (or other PCVs) and must not be planning to receive any PCV until the final analysis (day 181) is performed. Also, vaccination with PPSV23 should have occurred at least 1 year prior to screening, and the participant is not planning to receive the PPSV23 until the final analysis (day 181) is performed |
| Acute illness (this does not include minor illnesses such as diarrhea or mild upper respiratory tract infection) or temperature ≥38.0°C (100.4°F) within 24 hours prior to the administration of study vaccine |
| History of malignancy within 5 years before screening (exceptions are squamous and basal cell carcinomas of the skin and carcinoma in situ of the cervix, or malignancy, which is considered cured with minimal risk of recurrence) |
| Known allergies, hypersensitivity, or intolerance to ExPEC10V or its excipients |
| Known allergy or history of anaphylaxis or other serious adverse reactions to vaccines or vaccine products (including any of the constituents of the active control vaccines) |
| Contraindication to intramuscular injections and blood draws (eg, bleeding disorders) |
| Abnormal function of the immune system resulting from:  a. Clinical conditions (eg, autoimmune disease or immunodeficiency)  b. Chronic or recurrent use of systemic corticosteroids  *Note: Ocular, topical, or inhaled steroids are allowed*  c. Administration of antineoplastic and immunomodulating agents or radiotherapy |
| History of acute polyneuropathy (eg, Guillain-Barré syndrome) |
| History of chronic urticaria (recurrent hives), eczema, or atopic dermatitis |
| Received treatment with immunoglobulins in the 2 months or blood products in the 4 months before the planned administration of the study vaccine or has any plans to receive such treatment during the study |
| Received or plans to receive:  a. Licensed live attenuated vaccines - within 28 days before or after planned administration of the study vaccine  b. Other licensed (not live) vaccines - within 14 days before or after planned administration of the study vaccine |
| Received an investigational drug or used an invasive investigational medical device or received an investigational vaccine within 90 days before the planned administration of the study vaccine, or is currently enrolled or plans to participate in another investigational study until day 181 in this study  *Note:* *Participation in an observational clinical study is allowed with prior approval of the sponsor. During the long-term follow-up periods, participation in another investigational study is allowed only with prior approval of the sponsor* |
| History of an underlying clinically significant acute or uncontrolled chronic medical condition or physical examination findings for which, in the opinion of the investigator, participation would not be in the best interest of the participant (eg, compromise the well-being) or that could prevent, limit, or confound the protocol-specified assessments |
| Had major surgery (per the investigator’s judgment) within 4 weeks prior to randomization, or has surgery planned during the time the participant is expected to participate in the study or within 6 months after the last study vaccine administration  *Note: Participants with planned surgical procedures to be conducted under local anesthesia not judged as major by the investigator may participate* |
| Employee of the investigator or study site, with direct involvement in the proposed study or other studies under the direction of that investigator or study site, as well as family members of the employees or the investigator, or an employee of the sponsor |
| Chronic active hepatitis B or C infection, measured respectively by hepatitis B surface antigen test or by hepatitis C virus [HCV] antibody test; if positive, HCV RNA polymerase chain reaction test will be used to confirm active versus past HCV infection |
| Test positive for HIV type 1 or type 2 infection at screening |
| Cannot communicate reliably with the investigator |
| Who, in the opinion of the investigator, is unlikely to adhere to the requirements of the study or is unlikely to complete the vaccination and subsequent follow-up period(s) |
| Who has had major psychiatric illness and/or drug substance or alcohol abuse in the past 12 months , which in the investigator’s opinion would compromise the participant’s safety or compliance with the study procedure |

Abbreviations: ExPEC10V, extraintestinal pathogenic *E. coli* vaccine 10-valent; PCV, pneumococcal conjugate vaccine; PCV13, pneumococcal vaccine
13-valent, PPSV23, pneumococcal vaccine polyvalent.

**Supplementary Table 2. Cohort 1 Vaccination Schedule Plan**

|  | **Phase 1** | | | | | | **Phase 2a** | **Total** |
| --- | --- | --- | --- | --- | --- | --- | --- | --- |
|  | **Step 1** | **Step 2** | **Step 3** | **Step 4** | **Step 5** | **Step 6** | **Step 7** |  |
| **Vaccination on Day 1** | **Sentinel Participants (Low Dose)** | **Additional Participants (Low Dose)** | **Sentinel Participants (Medium Dose)** | **Additional Participants (Medium Dose)** | **Sentinel Participants (High Dose)** | **Additional Participants (High Dose)** | **Additional Phase 2a Participants** |  |
| Low-dose ExPEC10V | 2 | 18 |  |  |  |  | 80 | 100 |
| Medium-dose ExPEC10V |  |  | 2 | 18 |  |  | 80 | 100 |
| High-dose ExPEC10V |  |  |  |  | 2 | 18 | 80 | 100 |
| ExPEC4V | 1 | 3 | 1 | 3 | 1 | 3 | 40 | 52 |
| PCV13 | 1 | 3 | 1 | 3 | 1 | 3 | 40 | 52 |
| Total | 4 | 24 | 4 | 24 | 4 | 24 | 320 | 404 |

Abbreviations: ExPEC4V, extraintestinal pathogenic *Escherichia coli* vaccine 4-valent; ExPEC10V, extraintestinal pathogenic *E. coli* vaccine
10-valent; PCV13, pneumococcal vaccine 13-valent.

**Supplementary Table 3.** Multiplex ECL-Determined Immunoassay IgG Geometric Mean Titers and Geometric Mean Fold Increase From Baseline (Day 1)

| Serotype/  Day | ExPEC10V Low | | ExPEC10V Medium | | ExPEC10V High | | ExPEC4V | | PCV13 | |
| --- | --- | --- | --- | --- | --- | --- | --- | --- | --- | --- |
|  | **GMT**  **(95% CI)** | **GM FI (95%CI)** | **GMT**  **(95% CI)** | **GM FI (95%CI)** | **GMT**  **(95% CI)** | **GM FI (95%CI)** | **GMT**  **(95% CI)** | **GM FI (95%CI)** | **GMT**  **(95% CI)** | **GM FI (95%CI)** |
| O1A |  |  |  |  |  |  |  |  |  |  |
| Day 1 | 1250410.8 (1047711.7; 1492325.7) |  | 1193952.5 (978207.8; 1457279.9) |  | 1209227.0 (1006697.4; 1452502.0) |  | 1104178.0 (821844.7; 1483502.8) |  | 1310616.9 (1010870.8; 1699244.5) |  |
| Day 15 | 5350535.1 (4644976.0; 6163266.7) | 4.41 (3.690; 5.259) | 6358232.9 (5664895.6; 7136429.1) | 5.33 (4.429; 6.406) | 6421446.5 (5730738.4; 7195403.5) | 5.26 (4.381; 6.323) | 6930697.9 (5823442.2; 8248484.7) | 6.00 (4.472; 8.061) | 1606747.3 (1205014.3; 2142411.9) | 1.26 (1.086; 1.452) |
| Day 30 | 5112484.0 (4410187.9; 5926616.5) | 4.05 (3.466; 4.740) | 6188073.3 (5425498.1; 7057831.4) | 5.17 (4.284; 6.242) | 6383997.2 (5633938.0; 7233913.5) | 5.28 (4.405; 6.325) | 6070942.6 (4778712.5; 7712609.5) | 5.54 (4.082; 7.524) | 1794173.8 (1315930.3; 2446223.7) | 1.31 (1.129; 1.520) |
| Day 181 | 3583270.6 (2911726.7; 4409695.6) | 3.07 (2.598; 3.626) | 4537954.7 (3685416.2; 5587709.0) | 4.05 (3.272; 5.019) | 5046472.5 (4245661.9; 5998330.9) | 4.23 (3.442; 5.201) | 4468702.4 (3299074.4; 6053001.2) | 4.08 (3.055; 5.447) | 1523139.6 (1054776.8; 2199474.2) | 1.23 (1.045; 1.445) |
| Day 366 |  |  |  |  | 3934158.7 (3201033.1; 4835190.3) | 3.57 (2.893; 4.402) |  |  | 1605160.3 (1139469.6; 2261174.4) | 1.10 (0.969; 1.248) |
| O2 |  |  |  |  |  |  |  |  |  |  |
| Day 1 | 514069.4 (428397.0; 616875.0) |  | 465679.8 (393668.5; 550863.6) |  | 536303.6 (458544.7; 627248.6) |  | 479673.5 (365216.2; 630001.4) |  | 467863.4 (354874.1; 616827.7) |  |
| Day 15 | 4873016.8 (4116237.9; 5768931.0) | 9.54 (8.014; 11.360) | 4787891.5 (4034593.8; 5681837.2) | 10.05 (8.374; 12.056) | 6537864.9 (5861510.6; 7292263.1) | 12.31 (10.564; 14.334) | 6196015.3 (4955643.7; 7746845.5) | 12.80 (9.343; 17.530) | 447845.4 (343614.9; 583692.7) | 1.01 (0.953; 1.064) |
| Day 30 | 4748963.2 (4012632.8; 5620412.3) | 9.16 (7.714; 10.884) | 4270069.6 (3501400.5; 5207486.0) | 9.20 (7.551; 11.215) | 6330528.7 (5614246.1; 7138196.7) | 12.00 (10.318; 13.957) | 5413245.9 (4188560.3; 6996015.3) | 11.53 (8.447; 15.729) | 484833.8 (372182.7; 631581.8) | 1.05 (0.979; 1.116) |
| Day 181 | 2970482.1 (2354313.7; 3747913.3) | 6.14 (5.076; 7.438) | 3163734.9 (2474677.1; 4044656.4) | 6.67 (5.312; 8.382) | 4729234.1 (3894909.1; 5742279.2) | 9.38 (7.788; 11.309) | 4266510.5 (3102445.5; 5867343.2) | 9.04 (6.375; 12.817) | 490033.8 (367540.7; 653351.1) | 1.07 (0.990; 1.162) |
| Day 366 |  |  |  |  | 3550164.5 (2808076.4; 4488363.6) | 7.71 (6.279; 9.457) |  |  | 558977.9 (422941.4; 738769.7) | 1.22 (1.098; 1.348) |
| O4 | | | | | | | | | | |
| Day 1 | 462083.1 (395745.9; 539540.2) |  | 492816.2 (420242.9; 577922.5) |  | 451409.9 (398277.8; 511630.1) |  | 471249.5 (386304.4; 574873.2) |  | 429691.9 (352681.9; 523517.5) |  |
| Day 15 | 2906299.6 (2339299.2; 3610729.8) | 6.28 (5.103; 7.718) | 2778764.0 (2281609.7; 3384246.4) | 5.49 (4.528; 6.656) | 4140729.8 (3480255.9; 4926546.7) | 9.17 (7.610; 11.054) | 503269.8 (409184.0; 618989.3) | 1.06 (1.005; 1.127) | 545556.7 (415552.0; 716233.0) | 1.27 (1.081; 1.491) |
| Day 30 | 2849977.5 (2298957.5; 3533067.3) | 6.19 (4.989; 7.677) | 2286562.3 (1855540.8; 2817705.3) | 4.85 (4.007; 5.873) | 3835055.2 (3183368.8; 4620152.0) | 8.50 (6.996; 10.327) | 501717.6 (417216.6; 603333.0) | 1.08 (1.024; 1.146) | 585948.8 (448200.5; 766032.1) | 1.33 (1.115; 1.588) |
| Day 181 | 1719377.7 (1343456.0; 2200488.7) | 3.74 (2.949; 4.734) | 1464137.0 (1147541.0; 1868079.0) | 2.97 (2.478; 3.567) | 2063300.4 (1668018.7; 2552254.6) | 4.54 (3.753; 5.496) | 501991.4 (406178.0; 620406.3) | 1.04 (0.968; 1.125) | 549904.9 (410508.2; 736636.7) | 1.21 (1.032; 1.429) |
| Day 366 |  |  |  |  | 1429668.5 (1165397.8; 1753866.4) | 3.29 (2.762; 3.907) |  |  | 555656.1 (427063.9; 722968.3) | 1.20 (1.053; 1.356) |
| O6A |  |  |  |  |  |  |  |  |  |  |
| Day 1 | 1225843.7 (1066235.5; 1409344.2) |  | 1143877.4 (965179.1; 1355660.9) |  | 1137406.5 (996048.8; 1298825.4) |  | 1201196.6 (951495.1; 1516427.3) |  | 943745.8 (776652.7; 1146788.3) |  |
| Day 15 | 4349111.4 (3780753.5; 5002910.0) | 3.55 (3.039; 4.141) | 5150333.2 (4384440.1; 6050015.8) | 4.48 (3.731; 5.389) | 5834052.6 (5130881.4; 6633591.3) | 5.11 (4.408; 5.918) | 5051389.0 (4030377.7; 6331051.9) | 4.31 (3.336; 5.576) | 984170.4 (805275.1; 1202807.9) | 1.04 (0.990; 1.088) |
| Day 30 | 3914979.5 (3360214.3; 4561335.5) | 3.21 (2.745; 3.750) | 4675388.4 (3910364.2; 5590082.1) | 4.08 (3.355; 4.952) | 5328088.3 (4595271.6; 6177768.6) | 4.67 (3.988; 5.467) | 4648933.4 (3601807.2; 6000482.7) | 3.93 (2.991; 5.156) | 1018443.7 (813194.1; 1275498.2) | 1.01 (0.938; 1.088) |
| Day 181 | 2742527.1 (2289293.7; 3285491.5) | 2.10 (1.785; 2.477) | 3187945.6 (2581356.0; 3937077.1) | 2.90 (2.382; 3.532) | 3447910.8 (2852867.7; 4167066.4) | 3.15 (2.656; 3.738) | 3334516.2 (2533469.4; 4388842.5) | 2.69 (2.084; 3.480) | 958424.9 (745010.8; 1232973.1) | 0.93 (0.853; 1.006) |
| Day 366 |  |  |  |  | 2720316.5 (2256764.3; 3279084.9) | 2.54 (2.178; 2.958) |  |  | 1140758.7 (870845.9; 1494329.1) | 1.09 (0.988; 1.205) |
| O8 |  |  |  |  |  |  |  |  |  |  |
| Day 1 | 1548078.4 (1327332.5; 1805536.0) |  | 1732564.1 (1488898.1; 2016107.4) |  | 1555482.3 (1335617.8; 1811540.0) |  | 1557319.6 (1263249.1; 1919846.5) |  | 1339640.3 (1061371.9; 1690864.7) |  |
| Day 15 | 5082460.4 (4435261.0; 5824100.0) | 3.34 (2.892; 3.855) | 5605655.1 (4917150.4; 6390564.9) | 3.27 (2.791; 3.826) | 6071895.4 (5414031.1; 6809697.4) | 3.90 (3.362; 4.534) | 1738652.7 (1408103.9; 2146796.9) | 1.07 (1.033; 1.116) | 1391481.0 (1093370.5; 1770872.3) | 1.06 (0.999; 1.128) |
| Day 30 | 5443708.3 (4750360.8; 6238254.6) | 3.51 (3.065; 4.013) | 5717188.1 (4983597.2; 6558764.4) | 3.28 (2.810; 3.836) | 6287045.5 (5617980.2; 7035792.2) | 4.01 (3.437; 4.673) | 1684506.5 (1372618.1; 2067262.5) | 1.11 (1.054; 1.174) | 1549931.5 (1203616.5; 1995891.3) | 1.16 (1.040; 1.288) |
| Day 181 | 3792115.2 (3159463.7; 4551449.0) | 2.47 (2.155; 2.841) | 4351162.8 (3653295.9; 5182338.9) | 2.52 (2.135; 2.974) | 4752992.0 (4056882.5; 5568545.1) | 3.14 (2.673; 3.698) | 1757076.5 (1418223.5; 2176891.0) | 1.11 (0.990; 1.242) | 1637517.2 (1193309.1; 2247081.3) | 1.17 (1.022; 1.341) |
| Day 366 |  |  |  |  | 3623746.8 (3024857.5; 4341209.8) | 2.46 (2.110; 2.868) |  |  | 1430653.9 (1072253.4; 1908849.6) | 1.01 (0.892; 1.143) |
| O15 |  |  |  |  |  |  |  |  |  |  |
| Day 1 | 881437.1 (736740.8; 1054551.8) |  | 865326.5 (733883.5; 1020311.8) |  | 883763.0 (753753.2; 1036197.2) |  | 866342.0 (672364.6; 1116282.1) |  | 839725.1 (668057.1; 1055505.9) |  |
| Day 15 | 5151217.3 (4393448.3; 6039684.0) | 5.94 (4.966; 7.104) | 4635317.8 (3976055.4; 5403891.3) | 5.32 (4.468; 6.339) | 5414798.2 (4732722.1; 6195174.6) | 6.20 (5.248; 7.329) | 898897.3 (694569.8; 1163333.7) | 1.01 (0.971; 1.055) | 1202517.7 (893109.0; 1619118.0) | 1.46 (1.223; 1.753) |
| Day 30 | 4913158.9 (4214268.5; 5727952.5) | 5.43 (4.559; 6.458) | 4267640.2 (3590342.0; 5072707.0) | 4.99 (4.152; 6.003) | 5216657.6 (4496071.7; 6052732.0) | 5.78 (4.842; 6.910) | 900296.6 (691458.3; 1172209.6) | 1.03 (0.959; 1.098) | 1106266.6 (821060.3; 1490543.2) | 1.36 (1.184; 1.560) |
| Day 181 | 3171754.0 (2567192.7; 3918686.5) | 3.93 (3.213; 4.812) | 3172409.9 (2587224.3; 3889954.4) | 3.84 (3.160; 4.671) | 3306905.3 (2719748.6; 4020821.2) | 4.21 (3.470; 5.107) | 919201.8 (696966.2; 1212299.8) | 1.06 (0.931; 1.203) | 948722.9 (703688.5; 1279081.8) | 1.24 (1.071; 1.426) |
| Day 366 |  |  |  |  | 2758990.7 (2227724.4; 3416952.9) | 3.16 (2.615; 3.810) |  |  | 1026707.7 (748937.3; 1407499.4) | 1.24 (1.082; 1.423) |
| O16 |  |  |  |  |  |  |  |  |  |  |
| Day 1 | 857880.7 (725090.7; 1014989.2) |  | 853674.6 (740655.4; 983939.8) |  | 811314.4 (698903.0; 941806.1) |  | 685551.1 (580678.8; 809363.6) |  | 706903.3 (568193.4; 879475.6) |  |
| Day 15 | 4298055.1 (3664533.7; 5041099.1) | 5.04 (4.300; 5.912) | 3972656.6 (3378225.9; 4671682.9) | 4.59 (3.822; 5.502) | 5675420.6 (4957603.9; 6497170.6) | 7.02 (5.904; 8.342) | 759403.5 (619027.4; 931612.4) | 1.11 (0.987; 1.254) | 732969.6 (586564.3; 915917.3) | 1.08 (1.010; 1.155) |
| Day 30 | 4376021.6 (3720209.1; 5147443.3) | 5.05 (4.318; 5.912) | 3525973.6 (2932681.9; 4239290.3) | 3.98 (3.292; 4.805) | 5410106.5 (4684904.9; 6247565.9) | 6.60 (5.577; 7.802) | 723757.2 (621819.1; 842406.6) | 1.08 (1.016; 1.149) | 819745.0 (642584.7; 1045748.2) | 1.14 (1.059; 1.230) |
| Day 181 | 2952055.2 (2380469.2; 3660887.5) | 3.30 (2.752; 3.955) | 2527864.6 (2047268.5; 3121280.6) | 2.89 (2.361; 3.530) | 3713051.1 (3064737.4; 4498508.8) | 4.75 (3.969; 5.685) | 774950.8 (638278.5; 940888.3) | 1.06 (0.946; 1.182) | 720322.3 (537574.2; 965195.6) | 1.11 (1.024; 1.195) |
| Day 366 |  |  |  |  | 2784393.1 (2248058.4; 3448684.8) | 3.62 (3.025; 4.342) |  |  | 766620.1 (570562.4; 1030047.4) | 1.08 (0.949; 1.225) |
| O18A |  |  |  |  |  |  |  |  |  |  |
| Day 1 | 939614.7 (825189.6; 1069906.6) |  | 963410.7 (838467.8; 1106971.8) |  | 929906.5 (822791.8; 1050965.8) |  | 963103.2 (808065.2; 1147887.3) |  | 979084.2 (788765.0; 1215325.0) |  |
| Day 15 | 3532498.2 (2965791.1; 4207492.4) | 3.79 (3.220; 4.456) | 3585987.4 (3008355.6; 4274529.8) | 3.64 (3.043; 4.344) | 4522807.6 (3883477.9; 5267388.9) | 4.84 (4.132; 5.675) | 1015034.0 (840693.0; 1225529.4) | 1.04 (0.995; 1.087) | 1100583.1 (846592.3; 1430775.0) | 1.13 (1.027; 1.241) |
| Day 30 | 3500329.1 (2944423.4; 4161189.5) | 3.73 (3.158; 4.395) | 3317876.1 (2741547.6; 4015360.4) | 3.35 (2.777; 4.038) | 4518450.7 (3838422.1; 5318955.6) | 4.83 (4.067; 5.737) | 1006196.4 (830764.7; 1218673.8) | 1.05 (0.986; 1.116) | 1121774.9 (868211.3; 1449392.5) | 1.13 (1.004; 1.275) |
| Day 181 | 2219012.5 (1830470.2; 2690028.3) | 2.59 (2.192; 3.049) | 2376169.7 (1917314.2; 2944839.5) | 2.48 (2.061; 2.986) | 2586344.3 (2158196.6; 3099428.8) | 2.96 (2.481; 3.544) | 943359.0 (785332.4; 1133184.1) | 0.98 (0.911; 1.062) | 1025511.9 (773259.0; 1360054.9) | 1.10 (1.005; 1.206) |
| Day 366 |  |  |  |  | 2180993.4 (1813783.2; 2622547.1) | 2.37 (2.021; 2.778) |  |  | 1011099.1 (803648.4; 1272100.4) | 1.11 (1.009; 1.232) |
| O25B |  |  |  |  |  |  |  |  |  |  |
| Day 1 | 245540.8 (204971.5; 294139.8) |  | 240586.0 (200618.4; 288515.9) |  | 244026.3 (205790.7; 289366.1) |  | 190472.8 (150285.8; 241405.9) |  | 267582.0 (207517.9; 345031.0) |  |
| Day 15 | 1426991.8 (1113308.2; 1829058.4) | 5.80 (4.761; 7.071) | 2265970.3 (1766736.9; 2906273.8) | 9.51 (7.463; 12.126) | 2044257.9 (1617260.1; 2583994.0) | 8.24 (6.529; 10.394) | 2174362.2 (1533603.8; 3082837.4) | 11.02 (7.889; 15.388) | 267879.1 (204166.3; 351474.3) | 1.02 (0.932; 1.127) |
| Day 30 | 1345602.7 (1045822.6; 1731313.4) | 5.48 (4.481; 6.695) | 2067379.5 (1579663.8; 2705675.7) | 8.60 (6.697; 11.044) | 1720048.6 (1357130.9; 2180016.1) | 7.00 (5.596; 8.766) | 1651715.3 (1155425.4; 2361176.7) | 8.70 (6.293; 12.038) | 285650.4 (211348.9; 386073.0) | 1.03 (0.933; 1.141) |
| Day 181 | 831345.5 (623871.7; 1107816.5) | 3.61 (2.920; 4.468) | 1144537.9 (849494.3; 1542055.0) | 5.35 (4.133; 6.918) | 939742.9 (725097.2; 1217928.6) | 3.88 (3.114; 4.841) | 794017.4 (547313.1; 1151925.0) | 4.67 (3.387; 6.429) | 239952.5 (185411.5; 310537.2) | 0.99 (0.895; 1.104) |
| Day 366 |  |  |  |  | 744315.5 (587104.6; 943623.5) | 3.10 (2.573; 3.737) |  |  | 278650.7 (211316.5; 367440.3) | 1.16 (1.033; 1.293) |
| O75 |  |  |  |  |  |  |  |  |  |  |
| Day 1 | 1260552.6 (1058147.8; 1501674.0) |  | 1329257.4 (1126527.2; 1568471.0) |  | 1299454.7 (1114376.6; 1515270.9) |  | 1128587.7 (919661.0; 1384977.9) |  | 1373337.6 (1061821.6; 1776245.9) |  |
| Day 15 | 2932034.9 (2454267.7; 3502807.9) | 2.33 (2.013; 2.701) | 3107132.4 (2627869.3; 3673802.2) | 2.38 (2.055; 2.756) | 3963138.0 (3450884.5; 4551430.9) | 3.06 (2.622; 3.563) | 1336846.1 (1060936.4; 1684509.5) | 1.14 (1.026; 1.274) | 1363797.1 (1041800.4; 1785315.7) | 1.03 (0.945; 1.131) |
| Day 30 | 2827912.3 (2357677.6; 3391934.5) | 2.20 (1.915; 2.534) | 2640901.8 (2178489.5; 3201466.9) | 2.04 (1.749; 2.373) | 3569256.9 (3057254.3; 4167005.2) | 2.75 (2.344; 3.238) | 1180934.2 (941313.5; 1481552.7) | 1.06 (0.949; 1.177) | 1399568.8 (1063013.9; 1842678.4) | 1.03 (0.915; 1.149) |
| Day 181 | 1984426.0 (1622665.9; 2426837.6) | 1.59 (1.417; 1.790) | 1882478.8 (1529846.4; 2316393.5) | 1.54 (1.339; 1.776) | 2398218.7 (2000407.3; 2875141.0) | 2.07 (1.764; 2.430) | 1034083.5 (835708.8; 1279546.9) | 0.92 (0.829; 1.014) | 1159722.4 (858094.1; 1567375.9) | 0.92 (0.851; 0.984) |
| Day 366 |  |  |  |  | 1960676.9 (1639629.8; 2344586.6) | 1.71 (1.516; 1.931) |  |  | 1519723.2 (1099805.5; 2099970.1) | 1.05 (0.931; 1.182) |

Data presented are from the PPI analysis set. 95% CI for GMT and GM FI is based on the t-distribution.

Abbreviations: ECL, electrochemiluminescent-based assay; ExPEC4V, extraintestinal pathogenic *Escherichia coli* vaccine 4-valent; ExPEC10V, extraintestinal pathogenic *E. coli* vaccine 10-valent; GM FI, geometric mean fold increase; GMT, geometric mean titer; IgG, immunoglobulin G; PCV13, pneumococcal vaccine 13-valent; PPI, per protocol immunogenicity.

**Supplementary Table 4.** MOPA-Determined Functional Antibody Geometric Mean Titers and Geometric Mean Fold Increase From Baseline
(Day 1) to Day 15

| Serotype/  Day | ExPEC10V Low | | ExPEC10V Medium | | ExPEC10V High | | ExPEC4V | | PCV13 | |
| --- | --- | --- | --- | --- | --- | --- | --- | --- | --- | --- |
|  | **GMT**  **(95% CI)** | **GM FI (95%CI)** | **GMT**  **(95% CI)** | **GM FI (95%CI)** | **GMT**  **(95% CI)** | **GM FI (95%CI)** | **GMT**  **(95% CI)** | **GM FI (95%CI)** | **GMT**  **(95% CI)** | **GM FI (95%CI)** |
| O1A |  |  |  |  |  |  |  |  |  |  |
| Day 1 | 239.5 (195.6; 293.2) |  | 164.4 (133.2; 202.8) |  | 168.8 (136.5; 208.8) |  | 246.9 (176.3; 345.6) |  | 206.1 (158.0; 268.9) |  |
| Day 15 | 654.3 (512.4; 835.4) | 2.67 (2.103; 3.389) | 764.2 (590.3; 989.5) | 4.29 (3.372; 5.469) | 1129.5 (842.3; 1514.7) | 6.14 (4.546; 8.287) | 1377.3 (851.8; 2227.0) | 5.34 (3.533; 8.059) | 244.6 (177.9; 336.1) | 1.18 (0.934; 1.484) |
| Day 30 | 485.7 (383.6; 615.0) |  | 452.6 (343.0; 597.3) |  | 517.3 (383.9; 697.0) |  | 815.3 (545.7; 1217.9) |  | 300.8 (215.4; 420.1) |  |
| Day 181 | 369.0 (290.7; 468.3) |  | 249.1 (194.9; 318.4) |  | 291.5 (215.3; 394.7) |  | 466.6 (311.9; 698.0) |  | 254.3 (162.3; 398.4) |  |
| Day 366 |  |  |  |  | 261.2 (192.0; 355.4) |  |  |  | 284.2 (193.2; 417.9) |  |
| O2 |  |  |  |  |  |  |  |  |  |  |
| Day 1 | 368.3 (297.8; 455.4) |  | 294.3 (232.9; 371.8) |  | 399.2 (327.1; 487.2) |  | 401.9 (292.3; 552.8) |  | 347.4 (255.5; 472.4) |  |
| Day 15 | 5538.4 (3884.7; 7896.2) | 14.39 (10.285; 20.126) | 5359.8 (3689.3; 7786.7) | 17.78 (12.567; 25.168) | 12473.2 (9082.9; 17129.0) | 30.19 (22.504; 40.490) | 9629.4 (5960.7; 15555.9) | 22.96 (13.310; 39.589) | 363.5 (274.0; 482.3) | 1.06 (0.923; 1.228) |
| Day 30 | 2189.0 (1618.1; 2961.3) |  | 2533.1 (1768.1; 3629.1) |  | 4431.3 (3143.2; 6247.3) |  | 3748.8 (2391.6; 5876.4) |  | 270.4 (201.1; 363.6) |  |
| Day 181 | 1196.5 (869.4; 1646.7) |  | 1076.4 (777.7; 1489.8) |  | 1887.2 (1313.0; 2712.4) |  | 1687.3 (1057.5; 2692.1) |  | 246.7 (173.3; 351.3) |  |
| Day 366 |  |  |  |  | 1803.3 (1243.9; 2614.3) |  |  |  | 310.1 (222.4; 432.6) |  |
| O4 |  |  |  |  |  |  |  |  |  |  |
| Day 1 | 116.4 (96.3; 140.8) |  | 83.3 (67.8; 102.4) |  | 90.6 (77.9; 105.4) |  | 101.2 (72.4; 141.5) |  | 93.8 (72.9; 120.8) |  |
| Day 15 | 376.3 (278.6; 508.2) | 3.25 (2.489; 4.240) | 322.9 (239.7; 434.9) | 3.47 (2.723; 4.420) | 505.7 (374.6; 682.5) | 5.47 (4.169; 7.178) | 102.9 (74.1; 142.8) | 0.98 (0.852; 1.127) | 102.5 (79.3; 132.5) | 1.08 (0.917; 1.263) |
| Day 30 | 609.5 (470.2; 790.2) |  | 562.1 (431.7; 731.9) |  | 667.1 (518.0; 859.1) |  | 236.2 (177.7; 313.9) |  | 193.3 (152.8; 244.7) |  |
| Day 181 | 340.9 (259.8; 447.3) |  | 298.9 (231.5; 385.9) |  | 330.5 (263.7; 414.2) |  | 190.5 (140.6; 258.2) |  | 171.5 (124.8; 235.6) |  |
| Day 366 |  |  |  |  | 275.3 (216.8; 349.7) |  |  |  | 165.8 (125.8; 218.6) |  |
| O6A |  |  |  |  |  |  |  |  |  |  |
| Day 1 | 617.3 (478.4; 796.6) |  | 585.7 (445.6; 770.0) |  | 633.9 (511.5; 785.7) |  | 488.8 (331.7; 720.2) |  | 623.6 (458.6; 848.1) |  |
| Day 15 | 1495.9 (1133.1; 1974.8) | 2.30 (1.859; 2.858) | 2256.5 (1725.5; 2951.0) | 3.72 (2.740; 5.046) | 2217.2 (1690.8; 2907.6) | 3.54 (2.782; 4.510) | 2009.8 (1344.6; 3004.0) | 4.30 (2.840; 6.514) | 720.7 (513.8; 1010.9) | 1.18 (1.019; 1.368) |
| Day 30 | 740.5 (574.6; 954.4) |  | 1123.7 (864.6; 1460.4) |  | 994.7 (763.8; 1295.3) |  | 1067.7 (718.9; 1585.6) |  | 519.9 (339.3; 796.5) |  |
| Day 181 | 616.9 (469.9; 809.8) |  | 773.5 (580.5; 1030.7) |  | 599.4 (454.6; 790.3) |  | 623.3 (383.9; 1012.0) |  | 506.5 (280.3; 915.3) |  |
| Day 366 |  |  |  |  | 642.9 (489.7; 843.9) |  |  |  | 506.4 (302.5; 847.8) |  |
| O8 |  |  |  |  |  |  |  |  |  |  |
| Day 1 | 980.3 (774.4; 1241.0) |  | 722.4 (553.4; 943.1) |  | 907.6 (721.9; 1141.1) |  | 714.3 (535.9; 952.1) |  | 877.0 (679.3; 1132.2) |  |
| Day 15 | 1249.2 (979.3; 1593.5) | 1.26 (1.078; 1.468) | 921.5 (702.3; 1209.1) | 1.22 (1.107; 1.339) | 1037.6 (809.7; 1329.6) | 1.15 (0.995; 1.324) | 712.9 (527.1; 964.3) | 0.97 (0.801; 1.170) | 931.3 (720.7; 1203.4) | 1.07 (0.891; 1.282) |
| Day 30 |  |  |  |  |  |  |  |  |  |  |
| Day 181 |  |  |  |  |  |  |  |  |  |  |
| Day 366 |  |  |  |  |  |  |  |  |  |  |
| O15 |  |  |  |  |  |  |  |  |  |  |
| Day 1 | 613.0 (468.6; 801.8) |  | 458.4 (335.6; 626.3) |  | 519.6 (397.4; 679.5) |  | 690.9 (457.8; 1042.9) |  | 491.9 (344.0; 703.5) |  |
| Day 15 | 3095.1 (2194.3; 4365.6) | 4.91 (3.498; 6.884) | 3018.4 (2093.9; 4351.1) | 6.29 (4.285; 9.247) | 3658.5 (2528.2; 5294.1) | 6.87 (4.732; 9.980) | 650.2 (431.5; 979.8) | 0.95 (0.695; 1.289) | 831.9 (542.2; 1276.2) | 1.55 (1.076; 2.246) |
| Day 30 | 2423.3 (1767.9; 3321.7) |  | 2403.7 (1744.4; 3312.2) |  | 2658.9 (1931.1; 3660.8) |  | 477.8 (341.5; 668.4) |  | 527.5 (345.5; 805.4) |  |
| Day 181 | 1053.1 (724.1; 1531.6) |  | 1431.8 (1014.1; 2021.6) |  | 1110.1 (832.6; 1480.0) |  | 347.7 (240.4; 503.0) |  | 438.2 (282.6; 679.5) |  |
| Day 366 |  |  |  |  | 1064.8 (778.0; 1457.5) |  |  |  | 517.5 (325.3; 823.3) |  |
| O16 |  |  |  |  |  |  |  |  |  |  |
| Day 1 | 215.7 (170.6; 272.7) |  | 187.7 (150.4; 234.3) |  | 174.2 (140.7; 215.7) |  | 237.0 (168.9; 332.5) |  | 190.3 (146.1; 247.9) |  |
| Day 15 | 1532.2 (1092.6; 2148.8) | 6.58 (4.806; 9.018) | 1172.2 (822.4; 1670.8) | 5.86 (4.051; 8.485) | 1853.8 (1353.2; 2539.6) | 10.18 (7.527; 13.755) | 216.2 (147.0; 317.9) | 0.94 (0.795; 1.113) | 176.9 (134.4; 232.9) | 0.97 (0.821; 1.157) |
| Day 30 | 987.0 (727.8; 1338.5) |  | 1005.1 (701.4; 1440.3) |  | 1465.5 (1085.3; 1978.8) |  | 107.5 (74.6; 155.0) |  | 110.4 (78.1; 156.1) |  |
| Day 181 | 546.7 (386.5; 773.2) |  | 426.9 (298.6; 610.3) |  | 656.3 (474.2; 908.5) |  | 102.5 (73.3; 143.3) |  | 77.9 (51.6; 117.6) |  |
| Day 366 |  |  |  |  | 464.3 (320.7; 672.1) |  |  |  | 90.1 (59.5; 136.3) |  |
| O18A |  |  |  |  |  |  |  |  |  |  |
| Day 1 | 65.3 (51.8; 82.2) |  | 58.2 (44.8; 75.5) |  | 50.5 (40.7; 62.7) |  | 64.0 (43.8; 93.4) |  | 51.8 (38.8; 69.2) |  |
| Day 15 | 274.7 (202.2; 373.2) | 4.05 (3.148; 5.204) | 276.2 (205.0; 372.0) | 4.27 (3.192; 5.721) | 338.2 (245.3; 466.3) | 6.20 (4.645; 8.279) | 65.7 (44.6; 96.9) | 1.03 (0.869; 1.217) | 62.2 (42.3; 91.6) | 1.17 (0.941; 1.458) |
| Day 30 | 523.5 (412.6; 664.2) |  | 538.3 (402.8; 719.3) |  | 592.9 (451.5; 778.7) |  | 226.5 (155.7; 329.5) |  | 195.1 (141.5; 269.1) |  |
| Day 181 | 309.4 (242.6; 394.7) |  | 341.3 (255.5; 455.9) |  | 291.6 (221.1; 384.5) |  | 176.9 (123.3; 253.7) |  | 203.7 (142.8; 290.6) |  |
| Day 366 |  |  |  |  | 309.3 (239.9; 398.9) |  |  |  | 182.0 (134.9; 245.5) |  |
| O25B |  |  |  |  |  |  |  |  |  |  |
| Day 1 | 152.2 (117.8; 196.8) |  | 141.9 (107.9; 186.5) |  | 145.9 (115.8; 184.0) |  | 133.0 (97.9; 180.6) |  | 165.8 (119.1; 230.9) |  |
| Day 15 | 415.5 (318.4; 542.3) | 2.34 (1.883; 2.903) | 489.5 (373.3; 641.9) | 2.77 (2.155; 3.557) | 403.7 (312.4; 521.6) | 2.51 (2.033; 3.096) | 382.7 (268.7; 545.0) | 2.48 (1.830; 3.367) | 265.4 (173.1; 406.8) | 1.44 (1.040; 1.997) |
| Day 30 | 118.2 (91.1; 153.4) |  | 168.8 (122.2; 233.1) |  | 134.0 (103.5; 173.4) |  | 140.2 (97.0; 202.6) |  | 63.3 (<LLOQ; 96.5) |  |
| Day 181 | 75.4 (60.1; 94.6) |  | 84.3 (65.6; 108.3) |  | 74.1 (58.4; 94.0) |  | 67.8 (<LLOQ; 93.0) |  | <LLOQ (<LLOQ; 69.1) |  |
| Day 366 |  |  |  |  | 73.8 (<LLOQ; 96.8) |  |  |  | <LLOQ (<LLOQ; 80.5) |  |
| O75 |  |  |  |  |  |  |  |  |  |  |
| Day 1 | <LLOQ (<LLOQ; 39.0) |  | <LLOQ (<LLOQ; <LLOQ) |  | <LLOQ (<LLOQ; <LLOQ) |  | <LLOQ (<LLOQ; <LLOQ) |  | <LLOQ (<LLOQ; 39.0) |  |
| Day 15 | 84.0 (65.8; 107.1) | 1.92 (1.600; 2.299) | 58.0 (46.5; 72.3) | 1.69 (1.440; 1.991) | 91.7 (66.5; 126.5) | 2.58 (1.981; 3.355) | <LLOQ (<LLOQ; <LLOQ) | 0.97 (0.879; 1.064) | <LLOQ (<LLOQ; 45.3) | 1.03 (0.930; 1.133) |
| Day 30 | 221.2 (176.2; 277.7) |  | 148.5 (111.8; 197.2) |  | 246.4 (177.5; 342.1) |  | 71.9 (50.4; 102.7) |  | 97.6 (69.8; 136.5) |  |
| Day 181 | 121.7 (95.8; 154.8) |  | 72.4 (55.6; 94.1) |  | 103.1 (79.1; 134.5) |  | 40.3 (28.6; 56.9) |  | 55.1 (38.0; 80.1) |  |
| Day 366 |  |  |  |  | 81.1 (62.8; 104.7) |  |  |  | 90.4 (58.4; 140.0) |  |

Data presented are from the PPI analysis set. Clinical serum samples collected on days 1 and 15 were analyzed with a qualified MOPA and those from days 30, 181, and year 1 were analyzed with a validated assay; these data are not directly comparable. 95% CI for GMT and GM FI is based on the t-distribution. Abbreviations: ExPEC4V, extraintestinal pathogenic *Escherichia coli* vaccine 4-valent; ExPEC10V, extraintestinal pathogenic *E. coli* vaccine 10-valent;
GM FI, geometric mean fold increase; GMT, geometric mean titer; LLOQ, lower limit of quantification; MOPA, multiplex opsonophagocytic assay;
PCV13, pneumococcal vaccine 13-valent; PPI, per protocol immunogenicity.

**Supplementary Table 5.** **Vaccination Dose Selection by Assay Method and Analysis Set Based on Day 15 Immunogenicity Data**

|  | ECL-Based Immunogenicity | | MOPA | |
| --- | --- | --- | --- | --- |
| Serotype | Per Protocol Immunogenicity Analysis Set | Full Analysis Set | Per Protocol Immunogenicity Analysis Set | Full Analysis Set |
| O25B | **ExPEC4V**, ExPEC10V medium dose, ExPEC10V high dose | **ExPEC10V medium dose,** ExPEC10V high dose, ExPEC4V | **ExPEC10V medium dose,** ExPEC10V low dose, ExPEC10V high dose, ExPEC4V | **ExPEC10V medium dose,** ExPEC10V low dose, ExPEC10V high dose, ExPEC4V |
| O6A | **ExPEC10V high dose**, ExPEC10V medium dose, ExPEC4V | **ExPEC10V high dose**, ExPEC10V medium dose, ExPEC4V | **ExPEC4V**,  ExPEC10V medium dose, ExPEC10V high dose | **ExPEC4V**,  ExPEC10V medium dose, ExPEC10V high dose |
| O2 | **ExPEC10V high dose,** ExPEC4V | **ExPEC10V high dose,** ExPEC4V | **ExPEC10V high dose,** ExPEC4V | **ExPEC10V high dose,** ExPEC4V |
| O1A | **ExPEC4V,** ExPEC10V high dose | **ExPEC10V high dose,** ExPEC4V | **ExPEC10V high dose,** ExPEC4V | **ExPEC10V high dose,** ExPEC4V |
| O4 | **ExPEC10V high dose** | **ExPEC10V high dose** | **ExPEC10V high dose** | **ExPEC10V high dose** |
| O8 | **ExPEC10V high dose** | **ExPEC10V high dose** | **ExPEC10V high dose,** ExPEC4V | **ExPEC10V high dose,** ExPEC4V |
| O15 | **ExPEC10V high dose** | **ExPEC10V high dose** | **ExPEC10V high dose** | **ExPEC10V high dose** |
| O16 | **ExPEC10V high dose** | **ExPEC10V high dose** | **ExPEC10V high dose** | **ExPEC10V high dose** |
| O18A | **ExPEC10V high dose** | **ExPEC10V high dose** | **ExPEC10V high dose** | **ExPEC10V high dose** |
| O75 | **ExPEC10V high dose** | **ExPEC10V high dose** | **ExPEC10V high dose** | **ExPEC10V high dose** |
| Treatment selected | **ExPEC10V high dose** | **ExPEC10V high dose** | **ExPEC10V high dose** | **ExPEC10V high dose** |

The dose group with the highest least squares mean increase from baseline to day 15 per analysis set and per assay method is highlighted in bold. Other dose groups that are noninferior to this dose group based on the confidence limits are also shown in the table.

Abbreviations: ECL, electrochemiluminescent-based assay; ExPEC4V, extraintestinal pathogenic *Escherichia coli* vaccine 4-valent; ExPEC10V, extraintestinal pathogenic *E. coli* vaccine 10-valent; MOPA, multiplex opsonophagocytic assay.

**SUPPLEMENTARY FIGURES**

**Supplementary Figure 1**. Percentage and number of participants with solicited local (A) and systemic (B) adverse events by day


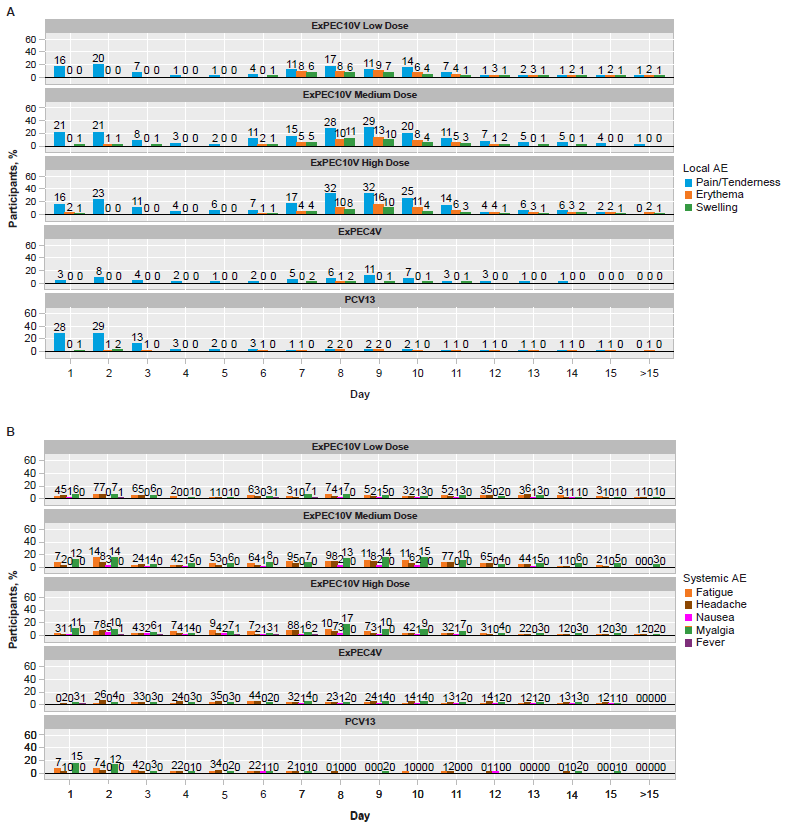
 Abbreviations: AE, adverse event; ExPEC4V, extraintestinal pathogenic *Escherichia coli* vaccine 4-valent; ExPEC10V, extraintestinal pathogenic *E. coli* vaccine 10-valent; PCV13, pneumococcal vaccine 13-valent.

**Supplementary Figure 2.** Correlation between ECL-based immunoassay and MOPA across serotypes


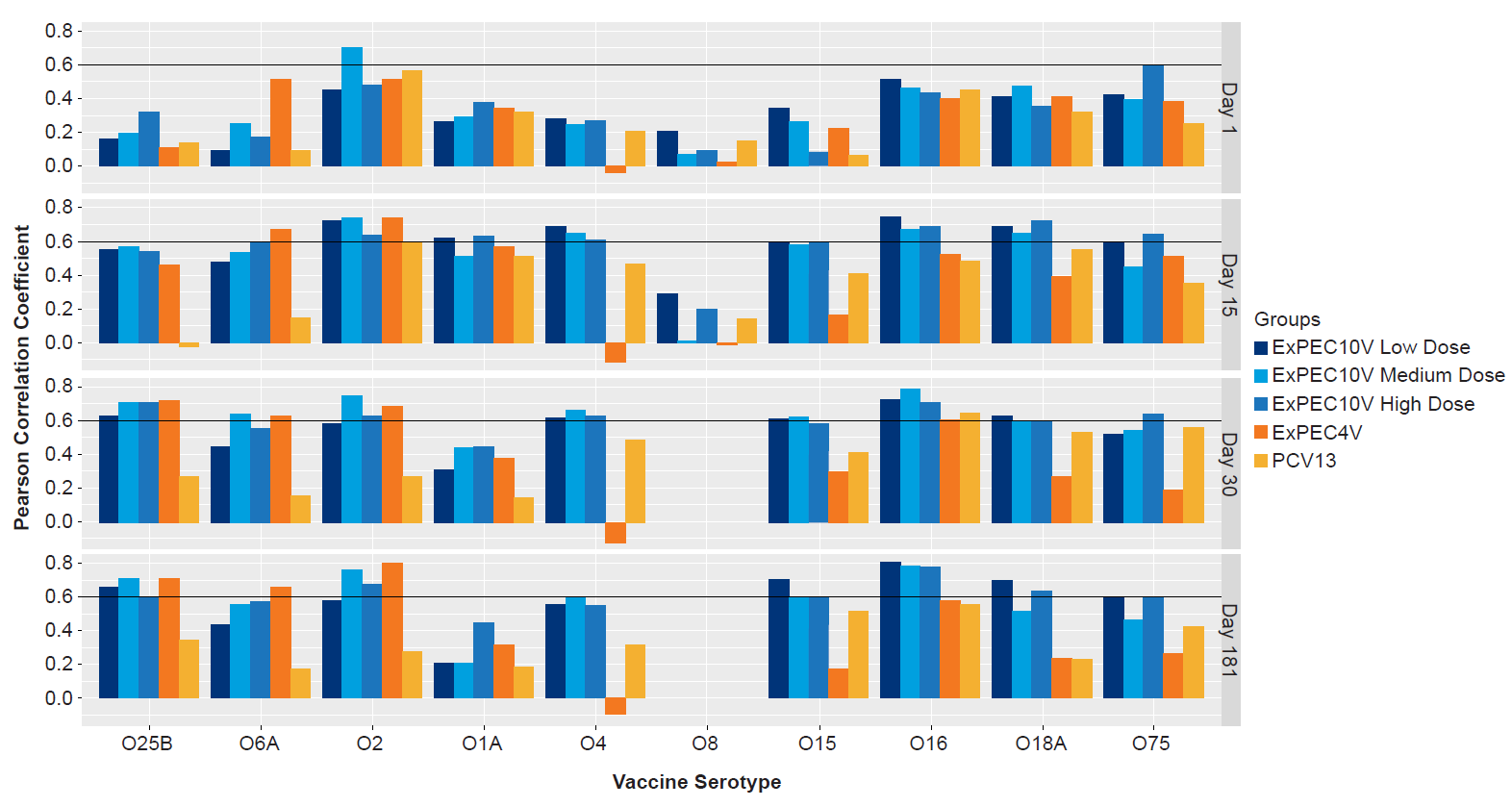


Horizontal line at 0.6 is arbitrary, intended to allow easy comparison of the magnitude of correlations across serotypes.

Abbreviations: ECL, electrochemiluminescent-based assay; ExPEC4V, extraintestinal pathogenic *Escherichia coli* vaccine 4-valent;
ExPEC10V, extraintestinal pathogenic *E. coli* vaccine 10-valent; MOPA, multiplex opsonophagocytic assay; PCV13, pneumococcal vaccine 13-valent.
